# A novel method of fabricating dynamic, heterogenous benchtop lateral and third ventricle phantoms from MRI of hydrocephalus patients: a verification and validation study

**DOI:** 10.1186/s12987-025-00742-w

**Published:** 2026-01-30

**Authors:** Blake Gura, Ahmad Faryami, Christopher Roberts, Carolyn A. Harris

**Affiliations:** 1 Department of Biomedical Engineering, Wayne State University, 818 W Hancock St, Detroit, MI 48201 USA; 2 Departmentof Chemical Engineering and Materials Science, Wayne State University, 5050 Anthony Wayne Dr, Detroit, MI 48202 USA; 3 Department of Chemical Engineering and Materials Science, Wayne State University, 6135 Woodward Avenue, Rm 3120, Detroit, MI 48202 USA

**Keywords:** Hydrocephalus, Benchtop model, Lateral and third ventricles, Ventricle morphology, Nominal-actual comparison, Ventricle model, Ventriculomegaly

## Abstract

**Background:**

Investigation of the failure of shunts used to treat hydrocephalus has been a topic of research for several decades, though it is difficult to understand the role of heterogeneous patient ventriculomegaly shifts over time. In this work, we present the design, verification, and validation of a novel benchtop model of the human lateral and third ventricles.

**Methods:**

3D models were rendered from MRI of pre-revision hydrocephalic patients (*n* = 6), printed in hollow High Impact Polystyrene (HIPS) molds, and injected with silicone rubber allowed to rotationally cure for three hours. Wall thickness of the ventricle models was measured via random point sampling (*n* = 300), assessing distribution of silicone rubber within the mold. One sample z-test was used to compare mean wall thickness to the target thickness of 1 mm. To visualize the inner volume post-hoc, expanding polyurethane foam was injected into ventricle models, creating negatives of the hollow molds. The negatives were 3D scanned and measured for frontal horn diameter, occipital horn diameter, Evan’s Index, Frontal-Occipital-Horn Ratio, and frontal horn volume. A single MRI was chosen for verification of repeatability (*n* = 11). Ventricular size was validated across models from patients (*n* = 6). Accuracy of ventricular expansion (indirect compliance assessment) was tested. For all tests, a confidence interval was set at 0.95 (α = 0.05).

**Results:**

The verification methods indicated that low variance of experimental clinical values were observed between ventricle model replicates. Nominal-actual comparison results consistently showed similar datapoint displacement densities between models. Validation showed that most ventricle geometries fell within 5% of MRI measurements. A strong positive correlation was observed between internal pressure and volume (*R*^*2*^ = 0.9932). Experimental Frontal Horn and Occipital Horn Diameter measurements compared to clinical observations showed anatomical accuracy.

**Conclusion:**

This study validates and verifies a model of the human ventricular system with arbitrarily chosen heterogeneous patient ventricles of varying morphologies and volumes. Paired with a pump, this model can be used to recapitulate cerebrospinal fluid flow. We show the recreation of patient-specific clinical lateral ventricle characteristics of size, shape, and static compliance control necessary to study the influence of these parameters on shunt function. The manufacturing process has the capacity to create accurate benchtop models of the lateral and third ventricles with geometric detail that should be refined over time with additive systems accounting for cranial viscoelastic compliance and varying material properties with elastic and shear moduli more similar to brain.

**Supplementary Information:**

The online version contains supplementary material available at 10.1186/s12987-025-00742-w.

## Background

Hydrocephalus is a neurological condition that generally describes an accumulation of excess cerebral spinal fluid (CSF) within the brain’s ventricular system, which can present in patients immediately following birth or much later in life. The reported incidence of congenital hydrocephalus varies greatly around the world, with estimates ranging from 5.9 per 10,000 births, to one in 500 births [1–3]. One of the complexities surrounding hydrocephalus is how to effectively treat patients: To date, the most common treatment method is the surgical placement of a ventriculoperitoneal (VP) shunt system that drains excess CSF from the patient’s ventricles into the peritoneal space [4]. Despite decades of ongoing research in improving shunt outcomes, the VP-shunt system has the highest failure rate of any implantable neurological device. Previous studies have shown that the failure of shunts is as high as 40% within the first two years following the surgery, with one cohort study finding that nearly 50% of all patients required one or more shunt revisions [5, 6]. Much of the research in hydrocephalus has focused on improving the long-term outcomes of patients with VP shunts through device and surgical improvements, along with understanding mechanisms of failure via endpoint analysis [7, 8]. Etiologies of ventricular catheter failure have been well characterized with proximal catheter obstruction being the leading cause, hypothesized to result from a foreign body response, over-drainage of the ventricles and ventricular wall contact, or greater influx of tissue aggregates leading to occlusion of the catheter tip holes. Other causes of failure include but are not limited to infection, disconnection, distal catheter obstruction, and valve failure [9–11]. The lack of improved outcomes for patients requiring VP shunts and the possibility of multiple revisions poses a significant burden on patients’ quality of life (QOL) and the U.S. healthcare system—many of the QOL impacts are understood to be shunt related, highlighting the need for improved clinical treatments [12, 13].

Previous studies have highlighted the importance of physical and chemical environmental conditions on implant successes and failures. This includes work in hydrocephalus where models are built to limit variables and test specific causal relationships, such as proximal catheter occlusion by proteins, cells, blood, over-drainage and under-drainage, and ventricular catheter mechanical readiness. Few of these benchtop models have utilized anatomically accurate chambers to represent the lateral and third ventricles. Those models that do attempt to recreate the complex anatomy of the ventricles are further hindered by limitations in modeling flexible materials capable of replicating tissue compliance and mimicking the dynamic ventricle environment. Finally, scalability is arduous and typically expensive, causing the remarkably heterogenous and often complex morphologies of patient’s ventricles to be simplified to hydrocephalic or healthy classifications [14–18]. With anatomically accurate ventricles, the dynamic relationship between the ventricular catheter location and its proximity to tissue masses may be better visualized and understood. Combined with adept patho-/physiologic flow setups, evidence from computational fluid dynamics models can be recapitulated and help understand over- and under-drainage impacts on mass flow, flow distribution, shear stress, and pressure.

This study presents the development, verification, and validation of a novel benchtop model of patient MRI-rendered lateral and third ventricles that are controllably compliant and accurately reflect the complex anatomy of the CSF system as it is relevant to the physical location of the proximal catheter of the VP shunt. These benchtop ventricle models have potential as a valuable research and clinical tool in investigating hydrocephalus. In future work, the system may be suitable to observe any changes in shunt function from catheter insertion approach, in shifts from over- or under-drainage, in varying ventricular catheter or valve type, and in modifying CSF pulsatility, viscosity, or the addition of protein or blood.

## Materials and methods

### Patient MRI and 3D model acquisition

High-resolution magnetic resonance imaging (MRI) data were obtained under IRB approved protocols at the University of Alabama at Birmingham, Wayne State University, and Washington University in St. Louis. Generally, data were deidentified, then sent to Wayne State University under a signed material transfer agreement or approved retrospective data share. Scans were collected from a subset of hydrocephalic pediatric patients, included for their moderate to enlarged ventricles undergoing clinical brain imaging pre-revision surgery using anatomical sequences secondary to diagnostic necessity. To attain scans, a GE Medical Systems MRI scanner was used with a 3.0T Signa HDxt system and 1.5T SIGNA Artist settings. Sagittal 3D T1-weighted BRAVO sequences were selected for their high spatial resolution and high contrast differentiation between CSF and surrounding brain tissue. Imaging parameters included isotropic voxel sizes (e.g., 0.9375 mm and 0.5469 mm), high in-plane matrix dimensions (up to 512 × 512), and slice thicknesses optimized for full-brain anatomical coverage. These sequences provided optimal input data for the segmentation of the ventricular system.

Image processing and segmentation were performed using both 3D Slicer and ITK-SNAP. In ITK-SNAP, the ventricular system was initially delineated using the active contour method, allowing for region of interest selection. Manual thresholding was subsequently applied to refine the segmentation and isolate the ventricular CSF from adjacent tissues. In 3D Slicer, the “Segment Editor” module was used to further edit the segmentation via iso-data classification followed by thresholding to enhance the contrast between CSF and surrounding brain structures. Ray-tracing techniques were applied to remove non-ventricular brain tissue, and visible artifacts were manually removed to optimize anatomical accuracy. Final segmentations were exported in the standard tessellation language (STL) format, and mesh surface refinement and rigidity adjustments were completed using Rhino 8, Spaceclaim, 3DS Max, and Autodesk Meshmixer for final closed-volume domain creation.

The frontal horn diameter (FHD), occipital horn diameter (OHD), and inner skull diameter (ISD) were obtained from the clinical MR imaging of each patient; clinical intracranial volume and CSF volume measurements were not included in patient profiles. Evan’s Index (EI) and frontal-occipital-horn-ratio (FOHR) were calculated for each patient from the clinical information in each MRI, using Eqs. (1) and (2), respectively. The EI and FOHR of each sample patient provided a relevant, simple, and consistent metric of comparison.

1 $$\:Eva{n}^{{\prime\:}}s\:Index=\:\frac{FHD}{ISD}$$

2 $$\:Frontal\:Occipital\:Horn\:Ratio=\:\frac{FHD+OHD}{2\times\:ISD}$$

**Fig. 1:**
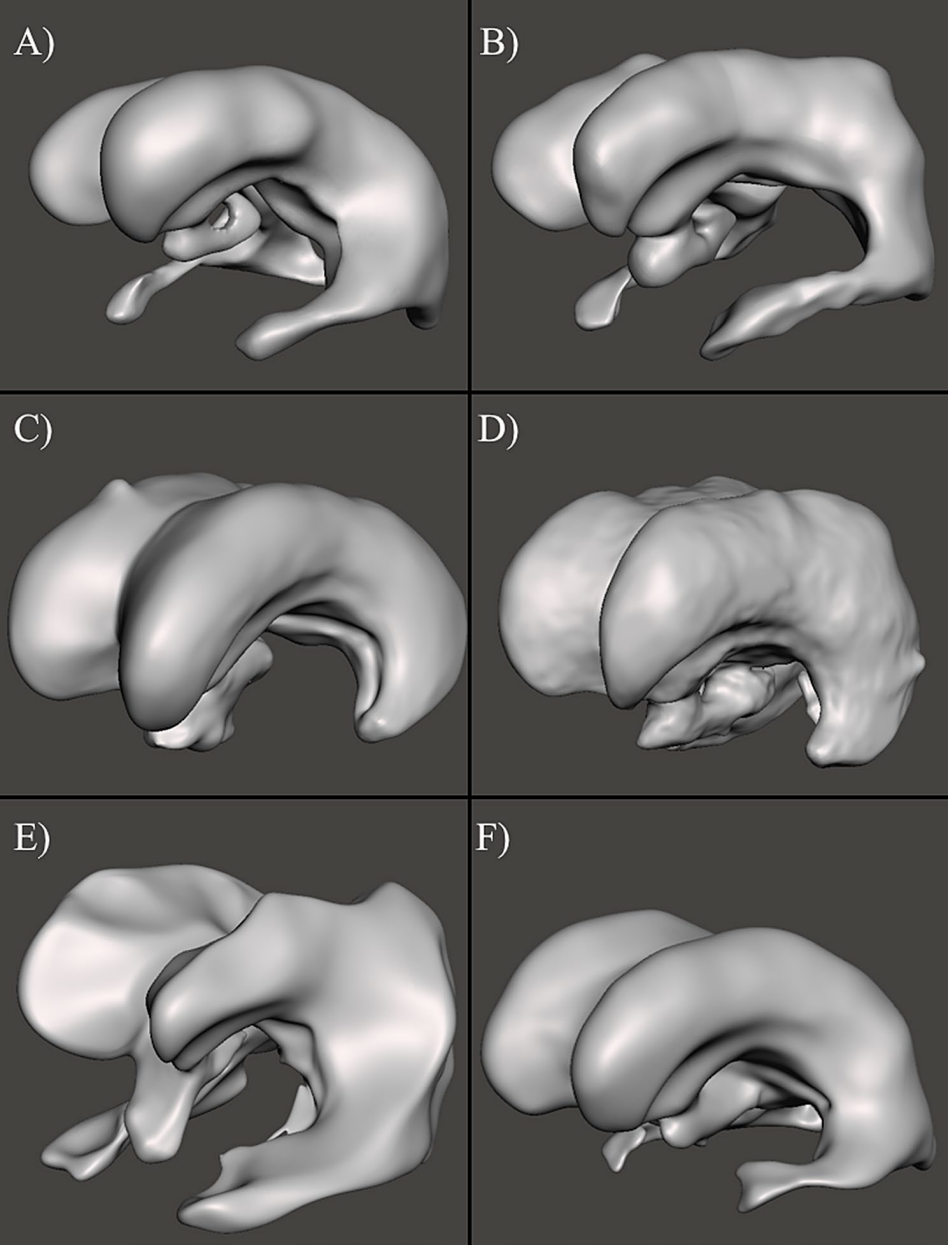
Front-left views of 3D ventricle models derived from patient MRI data. 3D models were loaded into Autodesk Meshmixer for imaging. Additional views of each model (lateral, cranial, caudal) are available in Supplementary Figs. 1–6. (**A**) *P-0* MRI model (**B**) *P-1* MRI model (**C**) *P-2* MRI model (**D**) *P-3* MRI model (**E**) *P-4* MRI model (**F**) *P-5 (II)* MRI model

**Fig. 2:**
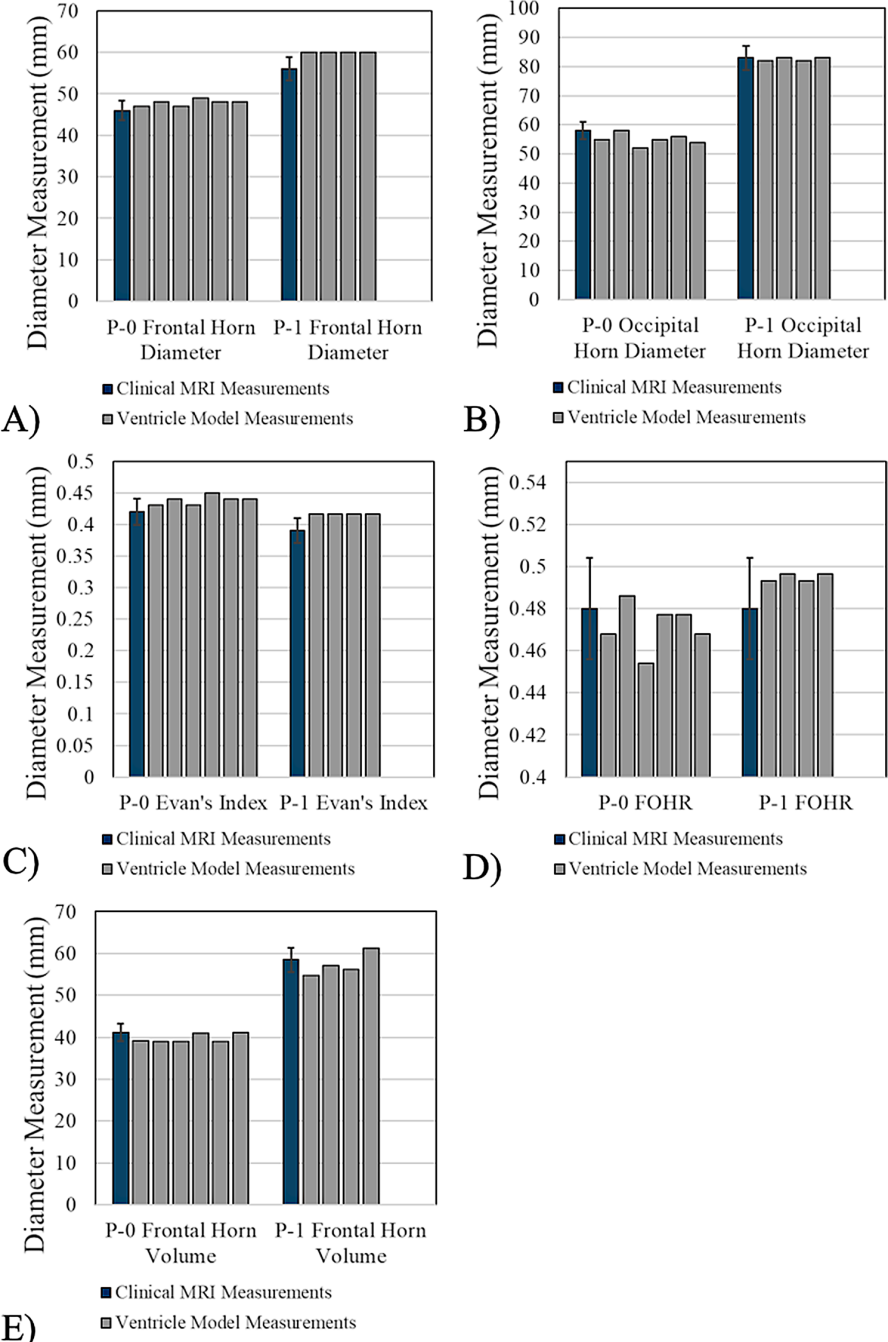
Comparison of *P-0* and *P-1* ventricle model measurements replicates to respective clinical values. (**A**) Frontal horn diameter comparison for all *P-0* and *P-1* model replicates to clinical values of *P-0* and *P-1* MR images (**B**) Occipital horn comparison for all model replicates to clinical MRI values (**C**) Evan’s Index comparison of all model replicates (**D**) FOHR comparison of all model replicates (**E**) Frontal horn volume comparison of all model replicates. Each bar in the graphs represents a different model replicate (*P-0-1 through P-0-*6; *P-1-1 through P-1-*5) Error bars present on the *Clinical MRI Measurements* bar within the graph represent a range of ± 5%. A range of values within ± 5% of clinical measurements was the expected/target range for experimental measurements to achieve. This convention is used for all subsequent figures comparing clinical values to experimental data

**Fig. 3:**
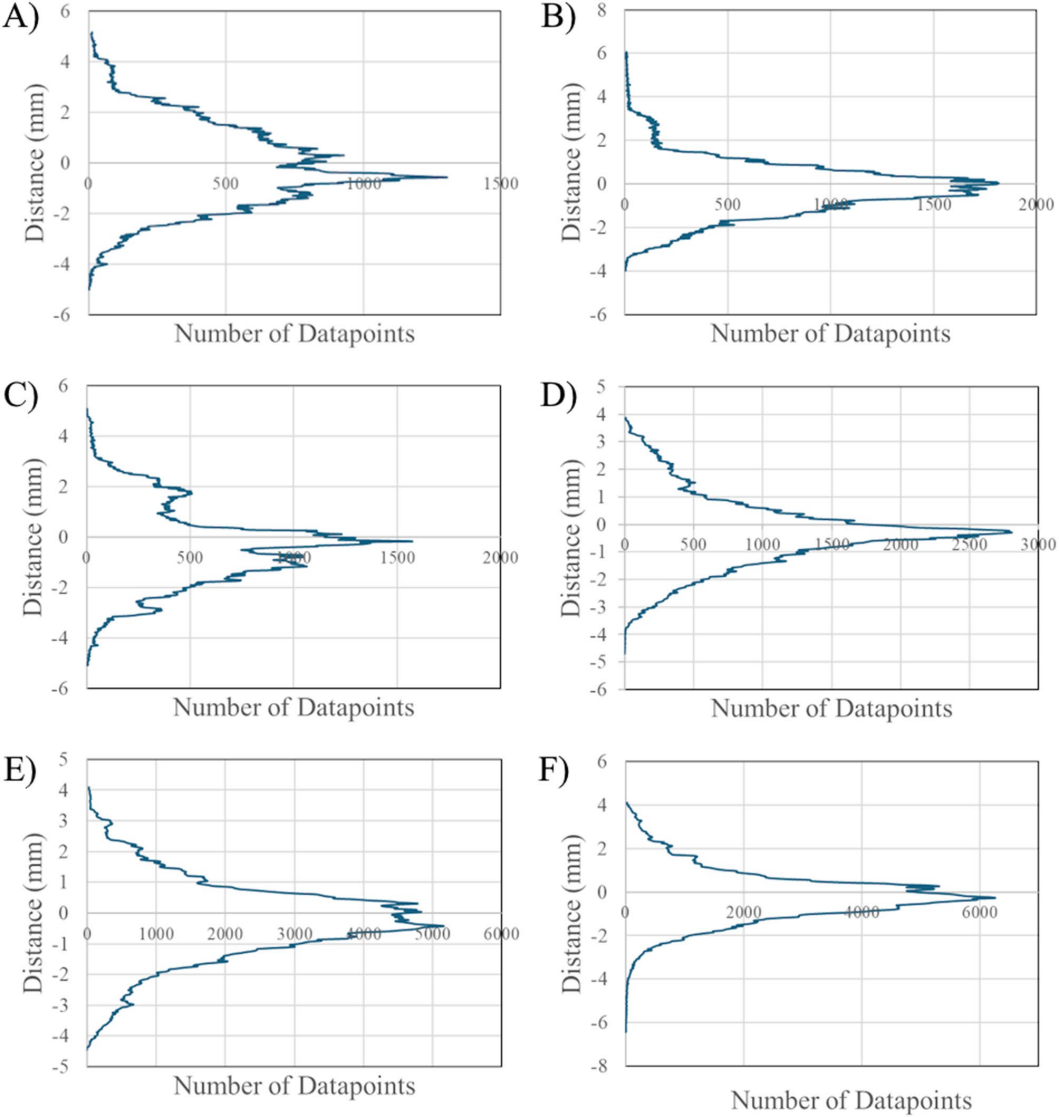
Nominal-actual comparison results comparing *P-0* model replicates to the *P-0* MRI. Datapoint densities representing the overall deviation of each *P-0* ventricle model replicate from the original MRI. Datapoints correspond to locations on the 3D-model mesh of the scanned ventricle models and then measured to the nearest related point on the mesh of the original MRI model. (**A**) Nominal-actual comparison displacements for *P-0-1* (**B**) displacement data for *P-0-2* (**C**) displacement data for *P-0-3* (**D**) displacement data for *P-0-4* (**E**) displacement data for *P-0-5* and (**F**) displacement data for *P-0-6*

**Fig. 4:**
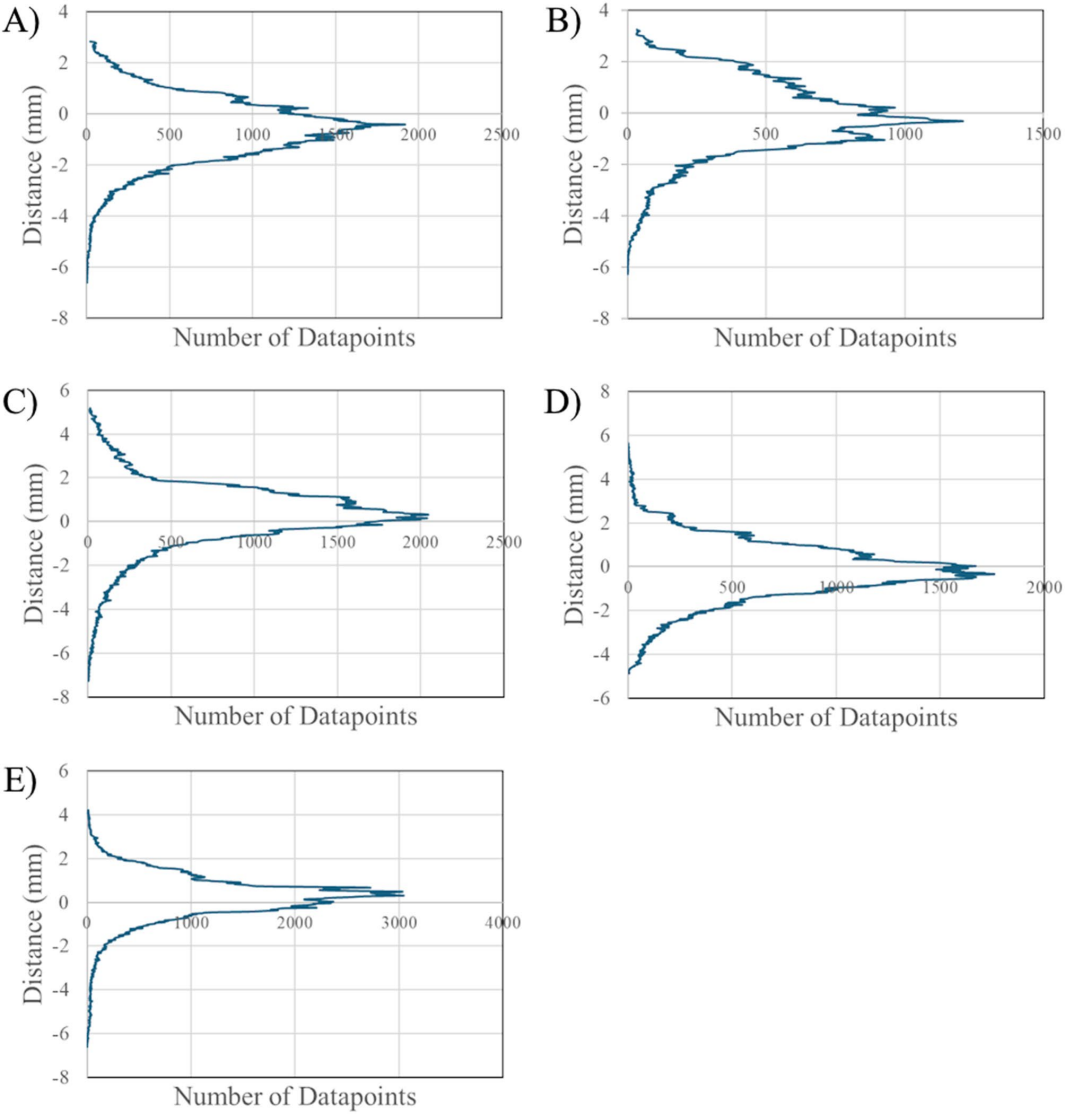
Nominal-actual comparison results comparing *P-1* model replicates to the *P-1* MRI. (**A**) Nominal-actual comparison displacements for *P-1-1* (**B**) displacement data for *P-1-2* (**C**) displacement data for *P-1-3* (**D**) displacement data for *P-1-4*,* and* (**E**) displacement data for *P-1-5*

**Fig. 5:**
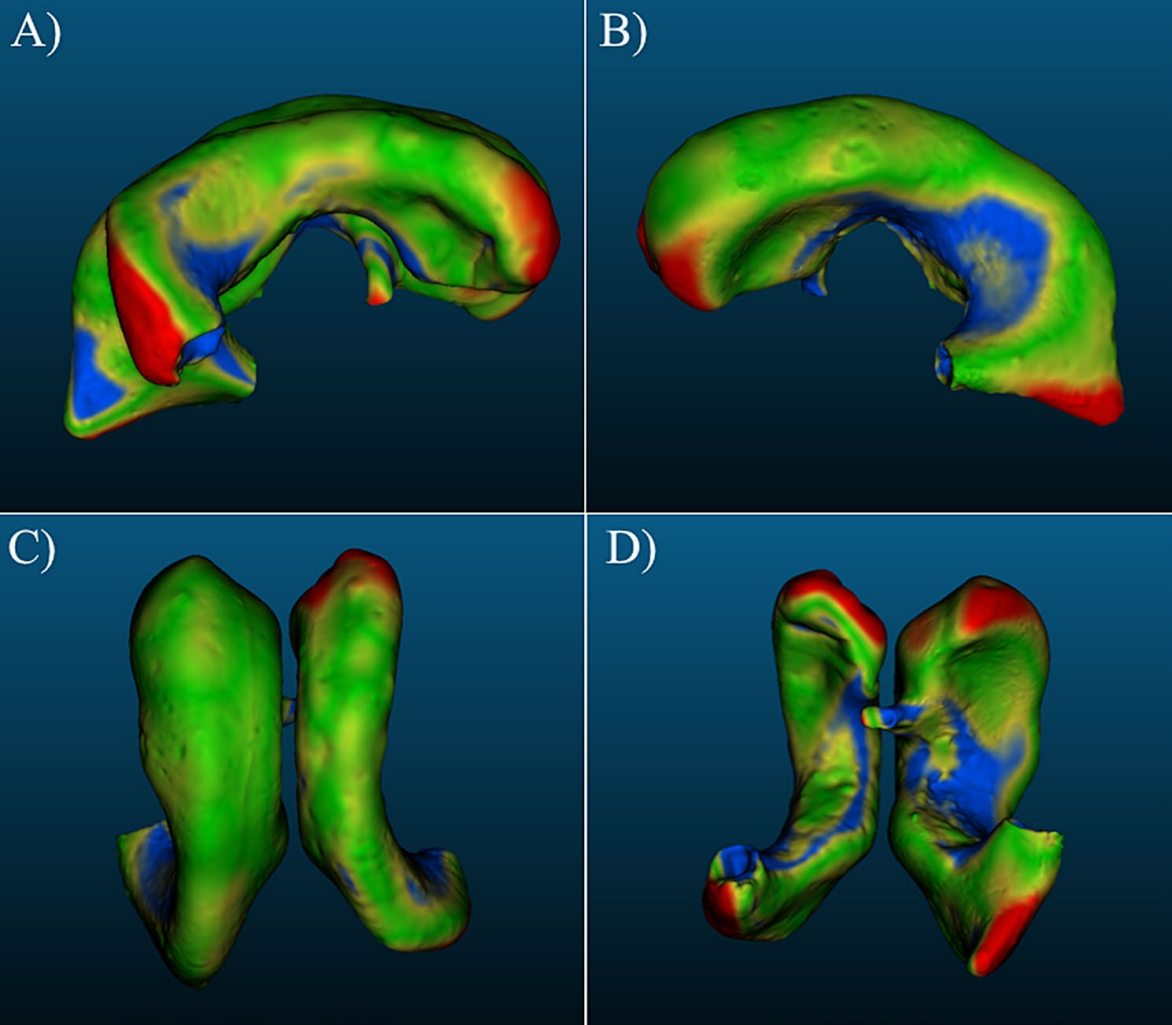
(**A-B**) Left and right lateral, (**C**) cranial (top-down) and (**D**) caudal (bottom-up) views of *P-0-6* 3D Scan and colormap *overlay.* Results from the nominal-actual comparison to the original *P-0* MRI model. 3D models generated by scanning a negative of the interior model space of the ventricle phantom were imported to CloudCompare, aligned with the 3D model of the corresponding MRI, and then software-driven calculations provide insights on how the 3D-scan and MRI geometries compare to each other. Coloring of different regions corresponds to point displacements from the MRI model. Green regions represent areas of the *P-0-6* model that deviate within ± 1 mm from the MRI, yellow regions represent areas of deviation within ± 2 mm of the MRI, blue regions represent areas of deviation <-2 mm, and red regions represent areas of deviation > 2 mm

**Fig. 6:**
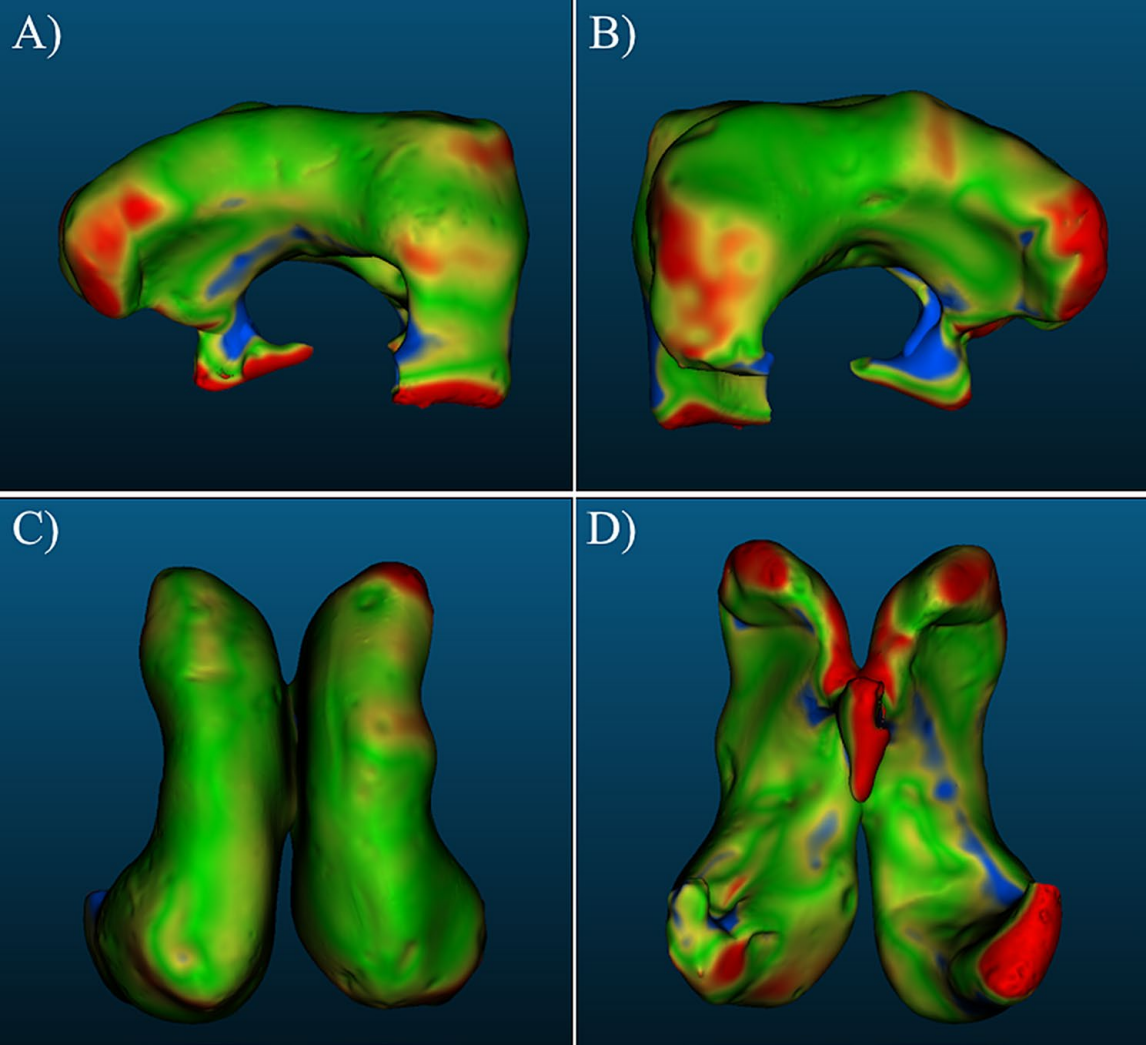
(**A-B**) Left and right lateral, (**C**) cranial (top-down) and (**D**) caudal (bottom-up) views of *P-1-5*. Multiple viewing angles of the *P-1-5* model with blue-yellow-green-red color map visualizing nominal-actual comparison results, completed with the same methods described for the *P-0* model replicates (Fig. 5)

**Fig. 7:**
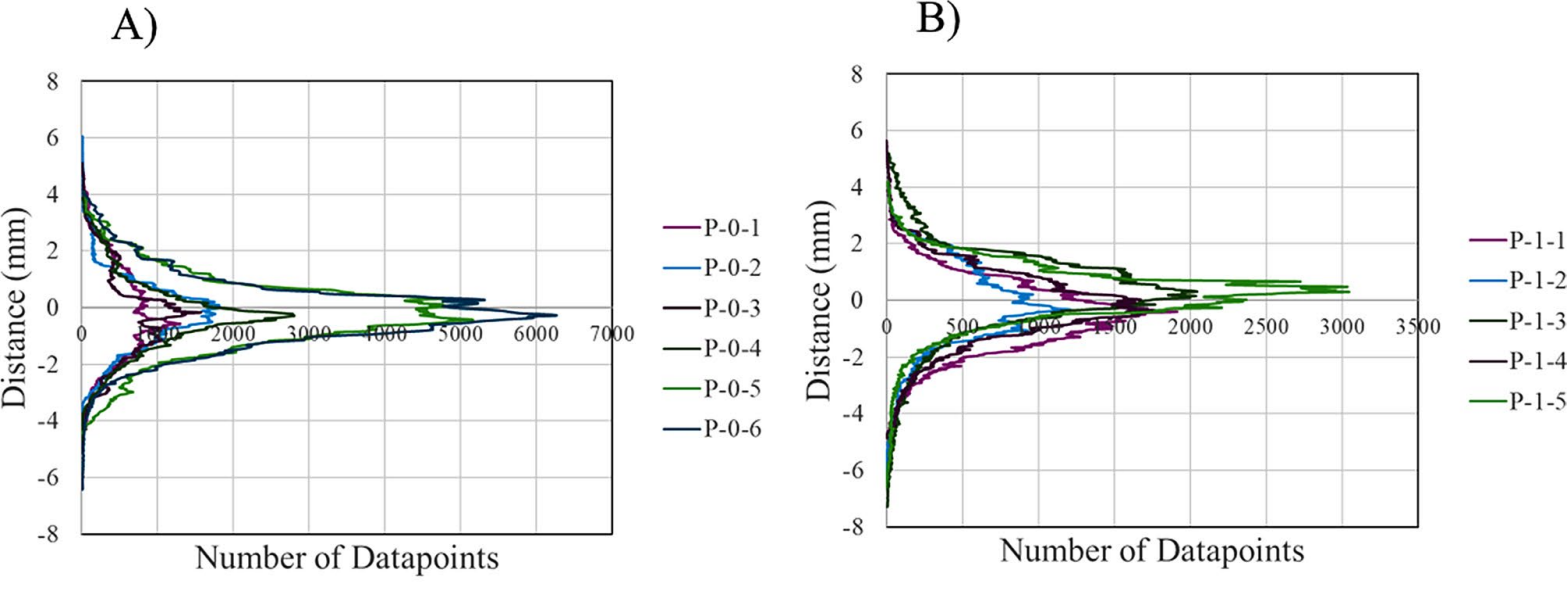
Overlayed datapoint distribution of nominal-actual comparisons for model replicates. The datapoint distribution for all (**A**) *P-0* and (**B**) *P-1* ventricle model replicates displayed within the same distance vs. datapoint plane to qualitatively assess the similarity and consistency of model replicates

Each model was randomly assigned an identifier following the format *P –* [0–5] to distinguish between different ventricles. In addition, three consecutive pre-revision scans from one patient were chosen to represent discrete stages in an individual patient. These scans were collected and labeled *P-5 (I)*,* P-5 (II)*, and *P-5 (III)*. Frontal horn measurements were not collected for *P-5 (I)*,* (III)* MRI models because they were not pertinent to their application in methods. MRI from six different patients with hydrocephalus were obtained and converted to high-quality 3-dimensional models (Fig. 1, S. Figures 1–6). In addition to the *P-5 (II)* (Fig. 1F), two additional 3D ventricle models were obtained from MR imaging of the same patient (S. Figure 7). Patient data including age, sex, time since last revision, comorbidities, and specific etiology was not collected. The EI, FOHR, and volume of each MRI were calculated using clinical scan measurements and in software (Table 1).

**Table 1:** Clinical measurements and software calculated frontal-horn volumes derived from MR images of hydrocephalic patients

Identifier	Frontal Horn Diameter (mm)	Occipital Horn Diameter (mm)	Inner Skull Diameter (mm)	Evan’s Index	FOHR	Frontal Horn Volume (mL)
*P-0*	46	58	109	0.42	0.48	40.68
*P-1*	56	83	144	0.39	0.48	58.44
*P-2*	46	84	136	0.34	0.48	84.12
*P-3*	42	75	121	0.35	0.48	144.00
*P-4*	53	84	124	0.43	0.55	53.02
*P-5 (I)*	74	100	151	0.49	0.57	N/A
*P-5 (II)*	65	93	149	0.44	0.53	104.06
*P-5 (III)*	55	84	138	0.40	0.50	N/A

### Ventricle model manufacturing process

A preliminary manufacturing process of our flexible silicone ventricle models from patient MRIs was briefly described in a previous study [19]. The process has since been updated significantly to produce high-quality ventricle phantoms. High-impact polystyrene (HIPS) molds were created of each patient’s ventricles from their 3D MRI models using a Creality Ender 3 S1-Pro Fused Deposition Modeling printer (Creality 3D Technology Co, Ltd, China). The HIPS molds were scaled up by a factor of 1.15 to account for internal volume loss through the manufacturing process and ensuring that the final interior space reflects the volume of the ventricles The 1.15 scaling factor was applied to each ventricle model to create the mold and is integral to the final accuracy of the ventricle model. This scaling factor was found to be the most adept for each model through a series of repetitive preliminary trials which involved manufacturing the ventricle model, completing a quantitative comparison to the original MRI, then adjusting accordingly, until the internal volume was as representative of the volume of the patient ventricles’ as possible with the current setup.

The HIPS ventricle molds endured a wash process to smoothen the interior surface of the mold space and reduce the likelihood of reduced ventricle model quality caused by interior mold imperfections. A 3 mm-diameter opening was made in the anterior wall of the frontal horn of both the left and right ventricles of the HIPS mold using a heated stencil at 200 °C. 5 mL of 100% acetone (Uniclean America Marine Chemicals, USA) were injected into each lateral ventricle and vigorously shaken for five minutes before being drained from the mold. The molds were drained and initially dried using pressurized air before being placed in the oven at 70 °C for 18 h To evaporate any remaining acetone.

Following the wash process, an additional 3 mm-diameter opening was made in each lateral wall of the occipital horns, allowing for the ventricle molds to be filled from four locations. Ecoflex™ 00–30 Platinum cure silicone rubber (Smooth-On, Inc., USA) was injected into the each opening in the left and right ventricular wall (Table 2); The volume of silicone rubber injected was determined to maintain a consistent wall thickness in each model. This was necessary because the geometry of each ventricle model contained unique morphologies and surface areas, introducing variation in how much silicone rubber was needed to achieve the same thickness. Immediately following the injection of silicone rubber into a mold, the mold was securely placed into the rotational molder to undergo biaxial rotation at 0.5 RPM with a 2:1 gear ratio between the primary and secondary rotational axes. Geared-down rotation about the secondary axis, as used in this process, is common in rotational molding applications since it often allows for more controlled and even material spread. After the first coat was complete, the filling and rotational molding was repeated for a second time to achieve the final wall thickness and developed ventricle phantom.

The HIPS molds were dissolved via acetone vapor deposition to retrieve the final ventricle models from each respective mold. The HIPS mold was placed on a stage in a desiccate chamber; 100mL of acetone was poured over it, then allowed to pool at the bottom. Over the course of 24 h, acetone vapor dissolved the HIPS mold causing it to separate from the silicone rubber and fall off, exposing the ventricle models. Once a significant portion of the model was exposed and the HIPS was mostly dissolved and pliable, the model was safely removed and cleaned of any residual HIPS.

**Table 2:** Material and handling properties of Ecoflex™ 00–30 platinum cure silicone rubber (Smooth-On, Inc., USA) per manufacturer report and additional material property reports [20, 21]

Property	Reported Value
Pre-cured mixed viscosity	3.0–6.1 Pa∙s
Pot life	45 min.
Shore Hardness	00–30
Elastic Modulus (G’)	0.1–0.5 MPa
Ultimate Tensile Strength	1.20–1.37 MPa
Elongation at Break	835% − 900%

### Model quantification

The overall spread of material/ thickness of the silicone rubber wall was measured to confirm quality and uniformity of potential expansion properties. After demolding, the silicone walls of the ventricle models were sliced with a scalpel along the sagittal plane through the third ventricle and transversely to create cranial and caudal wall sections. An electronic micrometer (Mitutoyo America Corporation, USA) was used to measure ten random points of the left and right ventricle walls for each ventricle phantom, and the mean ± standard deviation wall thickness (in millimeters) was calculated. The mean wall thickness indicated the overall silicone wall thickness of the model, and the standard deviation indicated how well the silicone rubber spread around the mold during the rotational molding process.

Three-dimensional scanning was used to visualize the interior space of the model (representing the volume of the lateral ventricles). A representation of the interior space of the ventricle models was created to enable quantification thereof and was not completed for any final ventricle models. An expanding polyurethane foam (Flex Foam-It III, Smooth On, Inc., USA) was injected into the HIPS molds following rotational molding, but prior to demolding. The expanding foam filled the space within the silicone model and created a negative of the hollow model; the interior, hollow space of the ventricle model is optimized to match that of the patient ventricles, while the silicone wall is analogous to brain tissue. After the expanding foam cured, demolding occurred as normal and the foam was removed from within the HIPS molds and silicone models. Prior to scanning, the foam ventricle models were spray-painted with a matte gray paint (Behr Process Corporation, USA) to improve the quality of the 3D scan. An Einscan SP V2 3D scanner (SHINING 3D, China) was used to scan the foam ventricle negatives and OEM provided software was used to ensure the 3D scans were properly meshed and exported to an STL format. The 3D models generated from the scanning process were imported into Autodesk Meshmixer to refine the scans and remove artifacts. The open-source metrology software CloudCompare was used to calculate their volumes, point-to-point measurements, and perform nominal-actual comparisons between the scanned negatives and their corresponding MRI model.

Experimentally similar FOHR and Evan’s Index values were calculated from point-to-point measurements of the 3D-scanned models at the same transverse slice of the MRI where the FHD and OHD were measured; clinical ISD measurements of patients were used for calculating EI and FOHR of the respective ventricle models. The transverse slice at which the FHD and OHD was determined by applying both an image overlay of the MR-image to the MRI-derived ventricle model and a normal map to locate curve maxima of the STL mesh. Once identified, the transverse slice at which measurements were taken was verified by taking point-to-point measurements of the MRI-derived model’s FHD and OHD and comparing them to clinical values. Once verified, the slice was marked and then used as a reference for the 3D-scanned models to take measurements at. The frontal horns of 3D scanned ventricle negatives were delimited from the rest of the STL and exported as a separate entity. The volume of the frontal horns of the ventricle models were calculated in software using a built-in mesh volume function based on the STL mesh data and the percentage difference was determined.

Nominal-actual comparison was used to compare the patient MRI and its respective 3D-scanned model to visually and quantitatively assess the difference in their 3D geometries and determine how accurately the ventricle models represent the ventricles of the patient. The STL meshes of both the MRI-derived model and the 3D-scanned model were imported into the metrology software and point matching was used to approximately align the meshes with each other. Once approximately aligned, point registering function was used with an RMS difference of $\:1\times\:1{0}^{-5}$ to create a very tight alignment between the two meshes. Mesh comparison was performed and then a normal map was applied to the 3D-scanned model that visualizes geometric differences between the MRI model and the scanned model. A blue-yellow-green-red colormap was used to represent certain displacement from the MRI model. Green regions of the colormap correspond to data points that fall within ± 1 mm displacement from the MRI, while yellow regions correspond to displacements of $\:\left[-2>d>-1,\:1<d<2\right]$ mm from the MRI. Blue and red regions of the colormap indicate displacements of <-2 mm and > 2 mm from the MRI, respectively. Positive displacement values represent regions that are larger than the MRI, while negative displacement values represent regions that are smaller than the MRI.

### Verification of ventricle models

Verification of our ventricle models involved manufacturing several models from the same patient profile and performing quantitative comparison between the replicates to assess the consistency and quality of the manufacturing methodology. Each model was quantified in software and experimental FOHR, Evan’s Index, and frontal horn volume values were derived. Nominal-actual comparison was used to geometrically compare each model to the patient MRI and histograms displaying datapoint displacement distribution were generated. The mean and standard deviation offset of our ventricle models were calculated along with the percentage of data that fell within 1σ, 2σ, ± 1 mm, and ± 2 mm. The *P-0* and *P-1* models were randomly chosen for verification. The multiple replicates of each model were differentiated by assigning an additional identifier to each one following the *P-0-[1–6]* and *P-1-[1–5]* format. The *P-0* model replicates were compared to the original *P-0* MRI model and among each other. Group mean, standard deviations, and variances were calculated for all metrics to assess variation of the models. The same methods were applied for all *P-1* model replicates.

### Validation of ventricle models

Validation of our flexible silicone rubber ventricle models involved completing direct quantitative comparisons of the models to the 3D model of the respective patient MRI. The six different ventricle models from patients with symptomatic hydrocephalus pre-revision were manufactured and compared to the corresponding MRI. Percentage differences of EI, FOHR, and front horn volumes between each MRI model and 3D-scanned model were calculated to assess how accurate the manufactured models are clinically. Histograms were generated from the nominal-actual comparison of each model. The mean ± standard deviation of the displacement data for each model was derived to determine the overall spread of the displacement and the average displacement relative to the target displacement of 0 mm. A mean displacement value approaching 0 mm indicated numerically that the MRI model and manufactured models are comparable to each other, while a lower standard deviation showed that mean displacement was not heavily influenced by large displacement values. The percentage of datapoints that fell within 1σ and 2σ displacements were calculated for each model to gauge the distribution of the displacement data, in conjunction with the mean for its skew. The percentage of datapoints within ± 1-, 2-, and 3-mm displacement was calculated from the nominal-actual comparisons to understand how the morphology of the manufactured models compares to the MRI model.

### Anatomically accurate shifts in volume – an indirect test for system compliance capacity

To characterize how the ventricle models change in relation to volume and internal pressure, compliance testing was done through a simple fluid circuit that allowed for in-flow and outflow through a ventricle model. Following model verification and validation, a series of three MRI across the same patient were chosen to test similarity in ventricular MRI volume and benchtop volume infused in progression as an indirect measure of the model’s capacity to mimic the development of ventriculomegaly and the reduction of ventricular size following a decrease in volume. As a precaution to leaks at the CSF inlet, a 16-gauge needle was used to puncture the ventricle model wall in the posterior-caudal portion of the occipital horns and filled with an additional 3 mL of silicone rubber. After injecting the additional material, ventricles were suspended in the oven at 70 °C for two hours until the silicone rubber was fully cured. A 16-gauge needle was once again used in the same fashion to create a puncture in the wall within the reinforced area, then a 10-gauge blunt syringe dispensing tip was press-fit through the puncture. The press-fit between the dispensing tip and the silicone rubber created a watertight and airtight seal at the interface and reliable inlet/outlet into the ventricle model were made.

The flow circuit began with a valve-controlled syringe channel from which water was pushed into the ventricle model through the right occipital horn inlet. Along this fluid channel, a pressure manometer (Manometer, RISEPRO, China) was attached via a three-way valve to measure the internal pressure of the ventricle model in *cmH*_*2*_*O* during the compliance testing. Outflow from the ventricle model occurred through the left occipital horn outlet and into a graduated container to measure the amount of volume drained during the compliance testing. The outlet was valve-controlled and included a check-valve to ensure fluid backflow or air leakage into the ventricle model did not occur. The ventricle model was primed via syringe vacuuming through the outlet to remove as much air as possible before filling with fluid. Priming was done again after filling with fluid by briefly submerging the model in a water bath to force expel any remaining air.

After completely priming, the outlet valve was closed and water was pushed into the ventricle model via syringe to bring the total water volume withing the model to 450 mL; this volume served as the initial volume for testing. From this initial volume, the outlet valve was opened and the water was allowed to drain from the model hydrostatically into the collection container at a flow rate of 7.12 mL/min. The ventricle model was drained until approximately 210 mL of water remained within the model. Pressure manometer readings and drainage volume readings were recorded at one-minute intervals for 33 min. The water volume remaining in the ventricle model was calculated from the initial volume and recorded drainage volumes. Relative bulk system compliance was defined as the change in volume (*ΔV*) relative to the change in pressure (*ΔP*), thus the equation $\:C=\frac{\varDelta\:V}{\varDelta\:P}\:mL/cm{H}_{2}O$ was used to calculate the relative bulk system compliance of the ventricle model from pressure and volume recordings. Storage and viscoelastic elastic properties of the system were not included and inherently different with silicone versus tissue. Lastly, failure testing of the model was completed by slowly pushing additional fluid into the ventricle model until either inlet seal or ventricle wall failure occurred. The maximum withstanding pressure and internal volume of the ventricle was recorded.

To make quantitative assessments and compare to the clinical values from the patient MR-images, approximate caliper (Electronic Digital Caliper, NEIKO, China) measurements of the frontal horns and occipital horns were taken when filled to specific, predetermined volumes. The ventricle model was filled with water to achieve the known clinical volume measurements of 198, 270, and 321 mL, derived from the *P-5 (I)*,* (II)*, and *(III)* MRI models, respectively, and FH and OH measurements were taken. These specific volumes chosen reflect the clinical volumes of the patients third and lateral ventricular volumes when the MR-images used were produced. Along with the clinical ISD measurements, the experimental EI and FOHR could be calculated for the ventricle model at each volume and compared to the clinical value.

### Statistical analysis

Our intent in this preliminary body of work was to determine if ventricles with heterogenous morphology can be repeatably manufactured from patient MRI using the described methods. It was not our intent to show that manufactured ventricles were not meaningfully different from the real-patient MRI population. Therefore, pair-wise comparisons were made over population-based statistical assessment. Statistical analyses were performed using JASP (University of Amsterdam, Netherlands). Group data for experimental measurements was reported as mean ± standard deviation, along with group variance. All nominal-actual comparison results are reported as mean ± standard deviation. A one-sample Z-test was used to compare model wall thickness to a target value within a large sample size. Students’ T-tests were used to compare experimental values to clinical values and assess statistical significance in difference. Linear regression was performed to correlate between time-sequenced variables, and the coefficient of determination (*R*^*2*^) was determined. For all tests, a confidence interval was set at 0.95 (α = 0.05).

## Results

Six different MR images of hydrocephalic patients were successfully sliced and converted to high quality, 3-dimensional models of lateral and third ventricles. All six ventricle models were further processed and successfully 3D-printed as hollow molds for rotational molding. Six replicate ventricle models of the *P-0* model and five replicates of the *P-1* model were manufactured for verification of the manufacturing process. One ventricle model was produced of the *P-2*,* P-3*, and *P-4* patient MRIs for model validation. Two ventricle models were produced of the *P-5* (II) patient MRI, one for model validation and one for compliance testing. A total of 16 ventricle models were manufactured and assessed. All ventricle models were successfully 3D scanned and quantified in software without major scanning errors or defects present.

The volume of silicone rubber injected into the left and right lateral ventricles was determined through repeat trials of each model to ensure consistent silicone rubber wall thickness (Table 3). Thickness measurements of the left and right ventricle model walls indicated that each model had a wall thickness approaching 1.00 ± 0.25 mm (Table 4). Between all models, there was a mean model wall thickness of 1.025 ± 0.193 mm. A one-sample z-test showed there was no significant difference between the mean wall thickness and the target thickness of 1.00 ± 0.25 mm (*p* = 0.08). Of the 300 randomly sampled points on the silicone rubber walls, 82% fell within 1σ.

**Table 3:** Injected silicone rubber volume into the lateral ventricles of the 3D printed molds (within an accuracy of ± 1 mL)

MRI Model	Left Ventricle Volume (mL ± 1 mL)	Right Ventricle Volume (mL ± 1 mL)
*P-0*	14	10.4
*P-1*	20	20
*P-2*	18	18
*P-3*	20	20
*P-4*	24	24
*P-5*	36	36

**Table 4:** Left and right mean silicone wall thickness for each ventricle model

Model Identifier	Mean left wall thickness ± SD (mm, *n* = 10)	Mean right wall thickness ± SD (mm, *n* = 10)
*P-0-1*	1.022 ± 0.30	1.034 ± 0.22
*P-0-2*	1.075 ± 0.20	1.032 ± 0.15
*P-0-3*	1.105 ± 0.14	1.007 ± 0.23
*P-0-4*	1.019 ± 0.07	1.007 ± 0.07
*P-0-5*	1.002 ± 0.17	1.036 ± 0.16
*P-0-6*	1.037 ± 0.16	1.024 ± 0.17
*P-1-1*	1.022 ± 0.30	1.034 ± 0.22
*P-1-2*	1.075 ± 0.20	1.032 ± 0.15
*P-1-3*	0.950 ± 0.17	1.001 ± 0.23
*P-1-4*	1.105 ± 0.13	1.007 ± 0.23
*P-1-5*	1.022 ± 0.16	0.951 ± 0.18
*P-4*	1.026 ± 0.180	0.871 ± 0.210
*P-5*	1.079 ± 0.130	1.027 ± 0.130
*P-6*	1.051 ± 0.160	1.069 ± 0.130
*P-8 (II)*	1.046 ± 0.180	1.035 ± 0.130

### Verification of ventricle models

The verification process of our ventricle models involved the manufacturing of multiple models derived from the same MRI model and cross comparing the models to ensure manufacturing consistency and replicability. The *P-0* and *P-1* models were chosen for completion of the verification process and each iteration was manufactured to the exact same specifications (*n* = 11). Clinical FHD, OHD, EI, FOHR, and frontal horn volumes were calculated for both *P-0* and *P-1* iterations. These values, in addition to data output of repeat measures of physical dimensions, represent total error propagation from all sources throughout analysis. Sources of potential variation/error throughout assessment include MRI voxel size, MRI segmentation thresholding, mesh processing volume loss, 3D printing software file conversion losses, scanner measurement error, and hand measurements using calipers.

For the *P-0* model, 83% of all measurements fell within ± 5% deviation from the original patient MRI model. The mean FHD and OHD measurements of all *P-0* models were 47.83 ± 0.75 mm (+ 4% from clinical FHD, σ^2^ = 0.567 mm^2^) and 55.00 ± 2.00 mm (-5% from clinical OHD), respectively, with FHD measurements of all models falling within 1σ, and the OHD measurement of 83% of models falling within 1σ (Fig. 2A-B). Greater variation was seen in OHD measurements compared to the FH diameters ranging from 52 mm (-10.3% from clinical) to 58 mm (same as clinical) and a sample variance of σ^2^ = 4 mm^2^. The Evan’s Index was consistently higher than the clinical value for each model being 0.44 ± 0.01 (+ 4% from clinical, σ^2^ < 0.0001) among all models with the Evan’s Index of all models falling within 1σ (Fig. 2C). Overall, the FOHR was consistently equal to or lower than the clinical value for each model being 0.47 ± 0.01 (-2% from clinical, σ^2^ = 0.0001) among all models, with the FOHR of 67% of models falling within 1σ (Fig. 2D). Similarly, the frontal horn volume calculations showed that the frontal horns of the *P-0* ventricle models were consistently equal to or less than what was calculated from the MRI-generated model, with a max deviation of -5%. Between all *P-0* models, there was a mean frontal horn volume of 39.71 ± 1.02 mL (-2% from clinical, σ^2^ = 1.044 mL^2^), with the volume of 67% models falling within 1σ (Fig. 2E). One sample student’s t-tests performed between the clinical parameters of the original *P-0* MRI and replicate ventricle models showed a statistically significant difference between the clinical Evan’s Index and group mean of the model’s (*p =* 0.002). However, differences between clinical and group mean FOHR measurements were not statistically significant (*p =* 0.286). The difference between the software-calculated frontal horn volume of the *P-0* MRI and model replicate volumes were found to not be statistically significant (*p =* 0.068).

For the *P-1* ventricle models, much tighter tolerances were observed between clinical metrics. 45% of all measurements fell within ± 5% deviation from the original patient MRI model, however, group variances of FHD, OHD, Evan’s Index, and FOHR were smaller compared to the *P-0* models. Of the five *P-1* models manufactured, only models *P-1-1*,* P-1- 3*,* P-1- 4*, and *P-1-5* could be assessed for clinical valuation due to the polyurethane foam negative of *P-1-*2 left ventricle not being obtained, thus could not be scanned. This resulted from an error in making the negative and was not a direct result of the manufacturing process itself. However, the 3D-scan model of the right ventricle for the *P-1-2* was still used for nominal-actual comparison. All usable *P-1* ventricle models had a measured FHD of 60 mm (+ 7% from clinical) (Fig. 2A). The mean OHD measurement of all models was calculated as 82.50 ± 0.58 mm (-0.60% from clinical, σ^2^ = 0.333 mm^2^), with all model measurements falling within 1σ (Fig. 2B). As a result of all models having the same FHD, Evan’s Index of each model were also the same, being 0.42 (+ 7% from clinical) (Fig. 2C). The FOHR of all models was marginally higher than the clinically observed value with a mean ratio of 0.49 ± 0.002 (+ 3% from clinical, σ^2^ < 0.0001) (Fig. 2D). The greatest variance in data was seen in the *P-1* models’ frontal horn volume with minimum and maximum volumes measuring 54.81 and 61.18 mL, respectively, and an overall variance of σ^2^ = 7.478 mL^2^. The mean frontal horn volume between models was 57.33 ± 2.73 mL (-2% from clinical) with 75% of all models falling within 1σ (Fig. 2E). One sample t-test indicated that the FOHR of the *P-1* MRI and ventricle model replicates were statistically significant (*p = 0.014)*, however the difference between frontal horn volume of the MRI and model replicates was not statistically significant (*p = 0.477)*. Comparison testing could not be done for Evan’s Index values in *P-1* model replicates due to all models having same calculated values.

Nominal-actual comparison data of the *P-0* model iterations and *P-1* model iterations are seen in Figs. 3 and 4, respectively. The histograms represent distribution densities of the displacement in millimeters of datapoints on a 3D-scanned ventricle model from the respective MRI model. The number of datapoints was software-determined and varied for each model. Color-coded histograms for each *P-*0 and *P-1* model are available in Supplemental Figs. 8–9, where the color-scale represents different distance intervals as defined in the methods and has been normalized to represent absolute distance from the respective MRI model, such that the same color will correspond to the same distance for each model. Left and right lateral, cranial, and caudal views of one iteration of the *P-0* and *P-1* model with nominal-actual comparison results are shown in Figs. 5 and 6, while additional images of each model are available in Supplemental Figs. 9, 10.

CAD comparisons done in CloudCompare between the *P-0* MRI model and each manufactured model; *P-0-1*,* P-0-2*,* P-0-3*,* P-0-4*,* P-0-5*,* and P-0-6*; indicated a small range of mean displacements from the original MRI. The smallest mean difference between a manufactured model and the MRI was seen in the *P-0-1* model, with a mean mesh displacement of -0.11 ± 1.56 mm from the MRI model. The largest difference was seen in the *P-0-3* model, with a mean mesh displacement of -0.32 ± 1.52 mm from the MRI. Between all models an overall displacement of -0.23 ± 1.34 mm was calculated. For all manufactured *P-0* ventricle models, greater than 68% of measured datapoints fell within 1σ of displacement measurements from the MRI. Similarly, greater than 95% of measured datapoint fell within 2σ of displacement measurements for all models. Greater variation was observed in the *P-1* model replicates; *P-1-1*,* P-1-2*,* P-1-3*,* P-1-4*, and *P-1-5*. The smallest deviation from the original MRI was observed in model *P-1-2* with a mean displacement of -0.15 ± 1.14 mm from the MRI, while the largest deviation was seen in model *P-1-1* with a mean displacement of -0.59 ± 1.21 mm from the MRI. Between all *P-1* model replicates, a mean displacement of -0.12 ± 1.28 mm from the original MRI was calculated. Similar uniformity was seen in each of the *P-1* replicates with 80% of the models having greater than 70% of measured datapoints fall within 1σ of displacement measurements from the MRI, with *P-1-2* only showing 61% of all datapoints being within 1σ of its mean displacement. However, only 40% of models showed greater than 95% of datapoints falling within 2σ; 60% of models showed a range of 90% to 94% of datapoints falling within 2σ. A largely Gaussian distribution was observed between all *P-0* and *P-1* replicates (Figs. 3, 4 and 7).

Merged datapoint densities for *P-0* replicates (Fig. 7A) and *P-1* replicates (Fig. 7B) qualitatively show within group comparison between the models, displaying how distribution of datapoint displacements for each model compare to each other. Notably, *P-0-5* and *P-0-6* have near overlapping distributions, while the distributions for the other models also exhibit near overlapping distributions with a greater concentration on the tails. The greater variation in *P-1* replicates is more clearly visualized by this qualitative comparison. The large disparities seen in the number of datapoints between each model replicate for both *P-0* and *P-1* models can be attributed to two factors: the exact mesh resolution of each model, a metric that changes through software processes at multiple steps throughout the methods. The lowest recorded datapoint count for *P-0* replicates was seen in the *P-0-1* model with 123,486 datapoints, while the highest was seen in the *P-0-5* model at 369,452. Similarly, the minimum and maximum datapoint counts for the *P-1* model replicates were 104,630 and 222,545 in the *P-1-2* and *P-1-5*, respectively.

### Validation of ventricle models

**Fig. 8:**
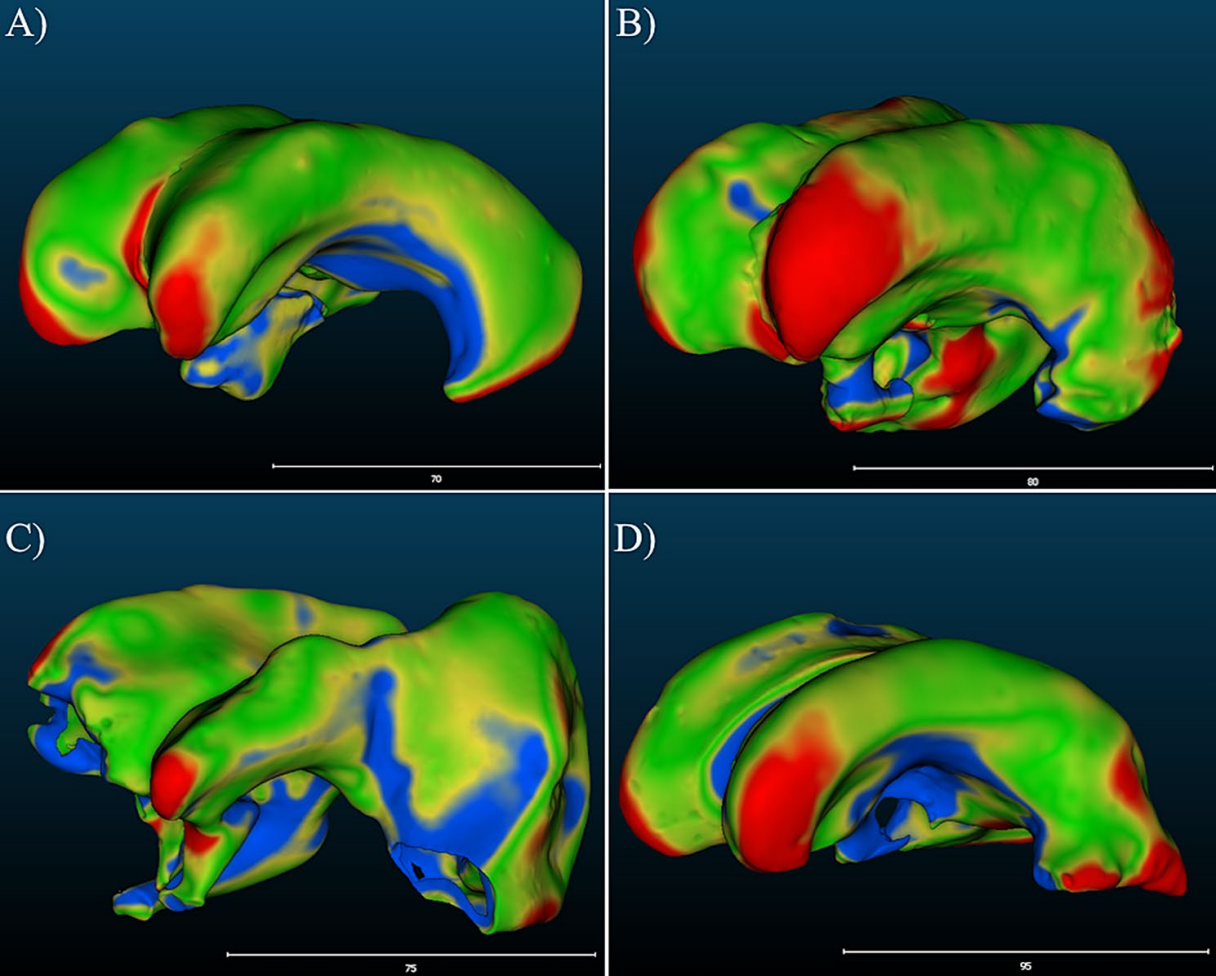
Top-left views of manufactured ventricle models with nominal-actual comparison color mapping. The scale in the bottom-left corner of each figure represents a distance scale in millimeters. **(A)** View of the *P-2* ventricle model **(B)** View of the *P-3* ventricle model **(C)** View of the *P-4* ventricle model. Notable portions that did not develop include part of the right frontal horn and left temporal horn. Further analysis revealed this to be symptom of the polyurethane foam, used to make the negative, not completely filling the inner volume of the ventricle model. The entire inner volume of the ventricle model was permeable to fluid up to the start of the temporal horns. **(D)** View of the *P-5* ventricle model. Underdevelopment of the temporal horns is present in all models due to limitations of the ventricle molds. The silicone rubber used to mold the ventricle models will fill the entire portion of the temporal horns within in the HIPS molds, however, the relatively high viscosity of the rubber creates a seal impermeable to fluid accessing all portions of the horns. In some cases, the total development of fluid-accessible temporal horns was observed

Validation of the ventricle models involved manufacturing of models derived from MRIs of multiple different patients and doing direct comparison of the manufactured models to the original MRI. In addition to the *P-0* and *P-1* models used for verification activities, four additional models were used (*n* = 6): *P-2* (Fig. 8A, S. Figure 11), *P-3* (Fig. 8B, S. Figure 12), *P-4* (Fig. 8C, S. Figure 13), and *P-5* (Fig. 8D, S. Figure 14). Frontal horn diameter, occipital horn diameter, Evan’s Index, FOHR, and frontal horn volumes were all successfully calculated and comparable to the clinical values derived from their corresponding MRI models. Nominal-actual comparison was successfully performed for all models as well.

**Fig. 9:**
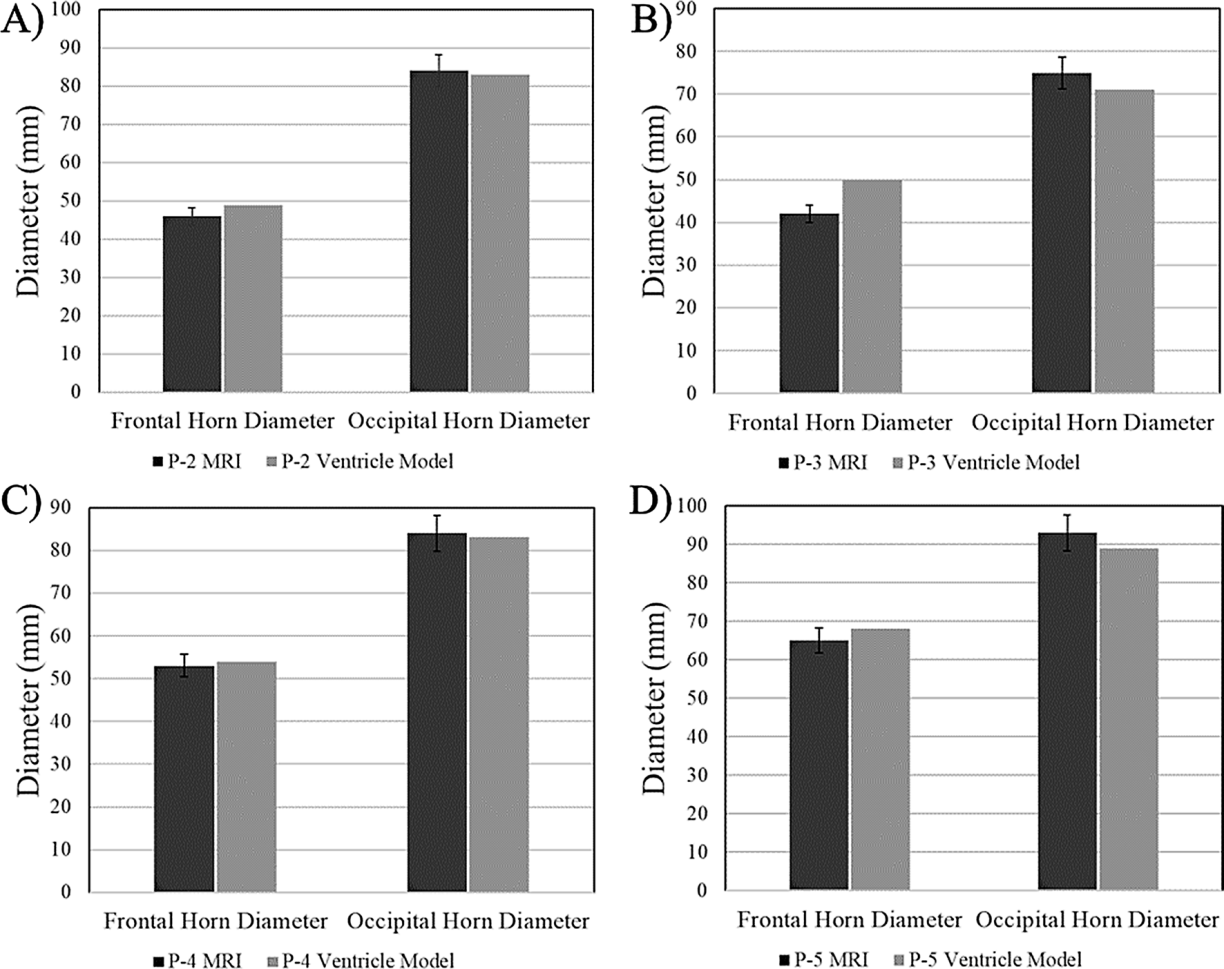
Comparison of experimental frontal and occipital horn measurements for ventricle models to clinical MRI data. Frontal and occipital horn measurements in millimeters of the (**A**) *P-2* ventricle model (**B**) *P-3* ventricle model (**C**) *P-4* ventricle model (**D**) *P-5* ventricle model to clinically derived values from their corresponding MRI. Error bars represent tolerances of ± 5%

**Fig. 10:**
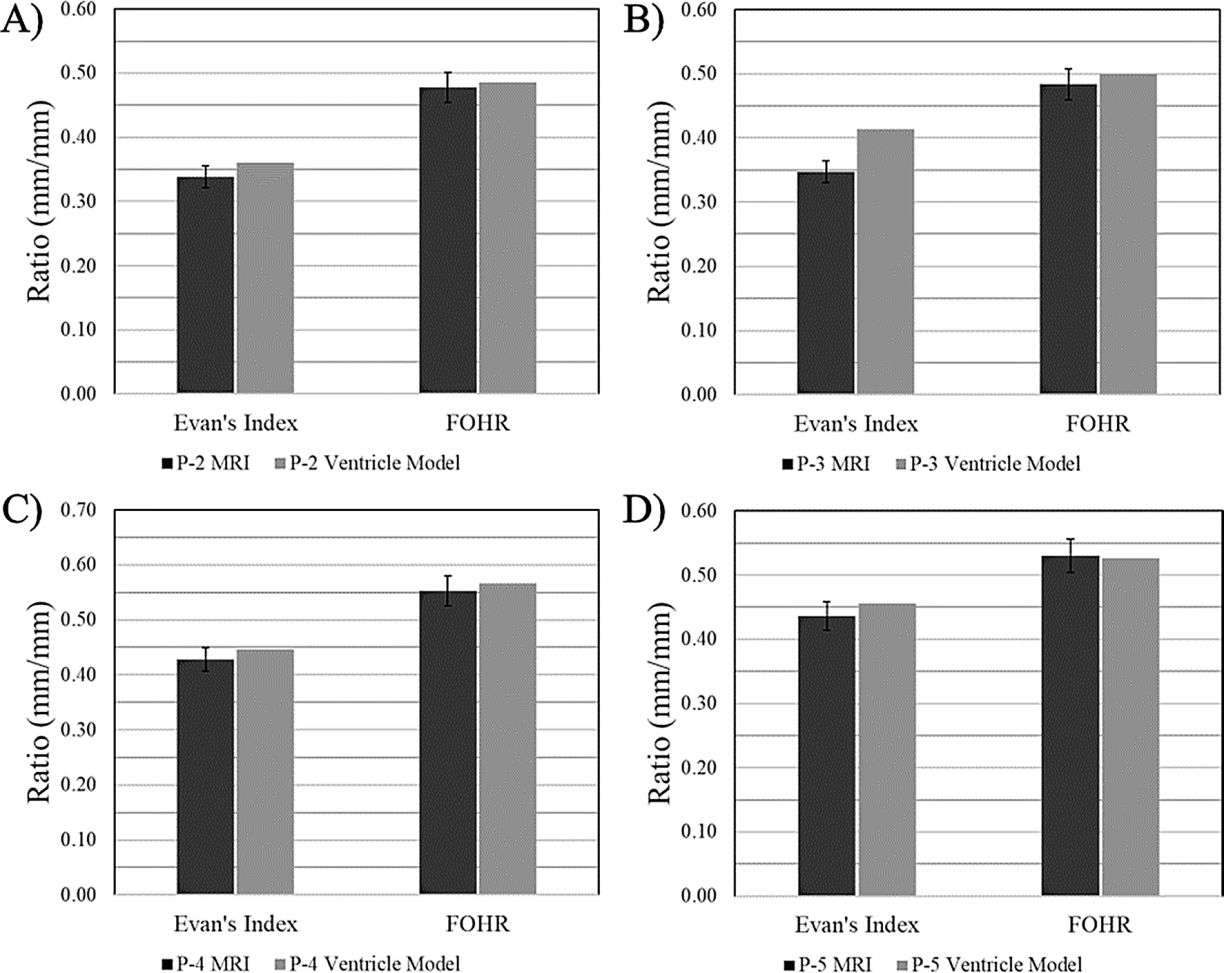
Comparison of Evan’s Index and FOHR values for ventricle models to clinical MRI data. Evan’s Index and FOHR (mm/mm) of the (**A**) *P-2* ventricle model (**B**) *P-3* ventricle model (**C**) *P-4* ventricle model (**D**) *P-5* ventricle model to clinically derived values from their corresponding MRI. Error bars represent tolerances of ± 5%

**Fig. 11:**
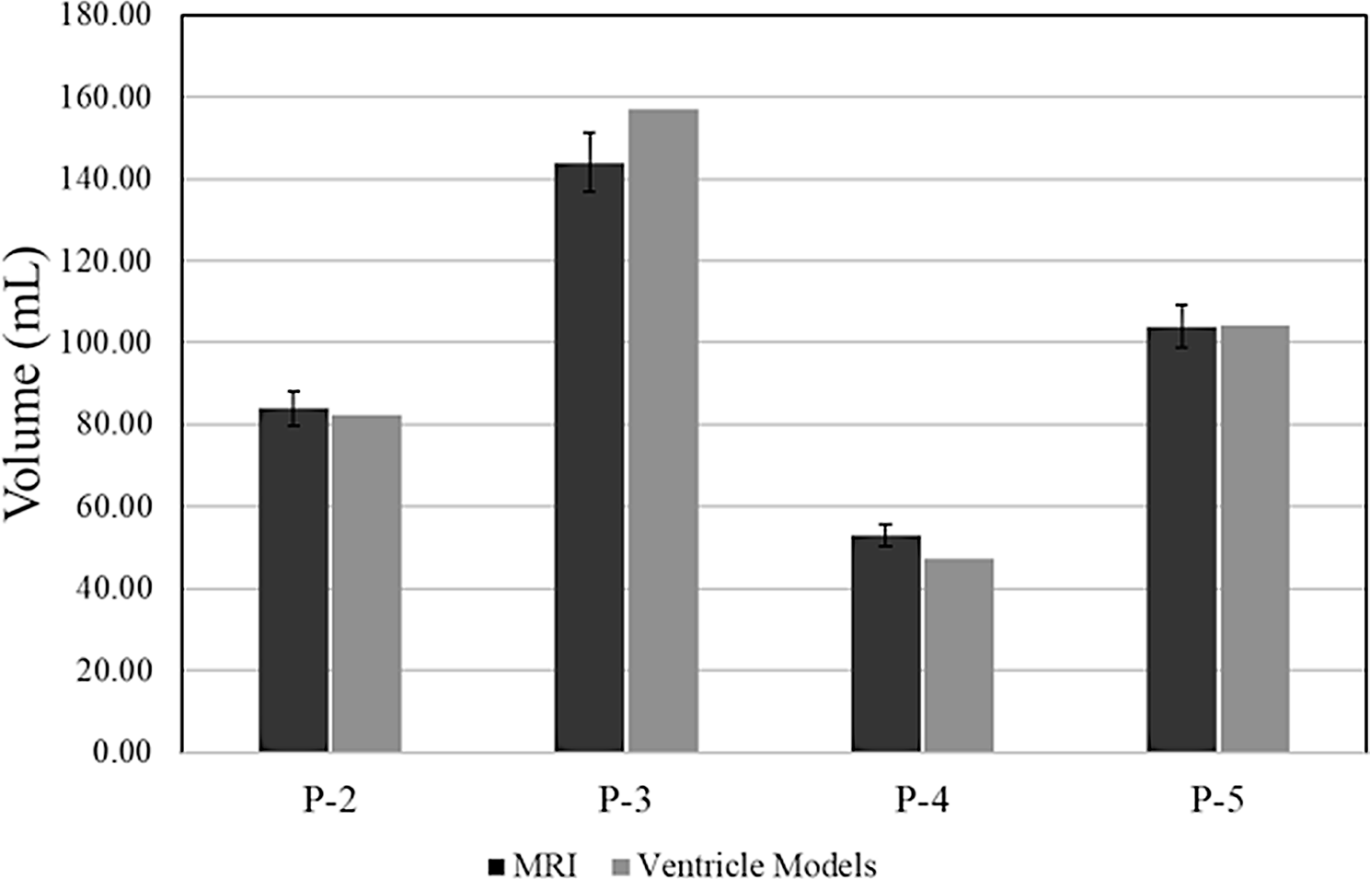
Comparison of frontal horn volume calculation of ventricle models to patient MRI model. Frontal horn volumes of each 3D scanned ventricle model, and its corresponding patient MRI were calculated in CloudCompare from the 3D mesh structure. Error bars represent tolerances of ± 5%

Column charts in Figs. 9, 10, 11 compare the clinical measurements of each ventricle model to their respective MRI. Frontal horn diameter measurements for the *P-2*,* P-4*, and *P-5* ventricle models differed from the clinical MRI value by no more than 3 mm. Occipital horn diameter measurements for all ventricle models differed from their clinical counterparts by no more than 4 mm, with all measurements falling within ± 5% of the clinical measurement (Fig. 9). Similarly, Evan’s index ratios of the *P-2*,* P-4*, and *P-5* ventricle models differed from clinically recorded values by no more than 0.02 (within ± 5% of clinical), while the *P-2* ventricle model had an Evan’s Index ratio 7% greater than the clinical value. The FOHR for all ventricle models had a difference from their respective MRI value no greater than 0.02 mm/mm and all values were within ± 5% of the clinical value (Fig. 10). Frontal horn volume calculations done in software showed that the *P-2* and *P-5* ventricle models were most similar to their MRI counterparts, showing a -2% and 0.10% difference in MRI and model volumes, respectively (Fig. 11). Larger discrepancies were seen in the *P-3* and *P-4* ventricle models, with the frontal horn volumes of both showing a 9% and − 10% difference from the volume of the MRI model, respectively. *P-2* and *P-5* ventricle models showed a volume difference from the MRI model of no greater than 1.5 mL, while the *P-3* ventricle model volume was 13 mL greater than the MRI, and the *P-4* model volume was 5.54 mL less than the MRI.

**Fig. 12:**
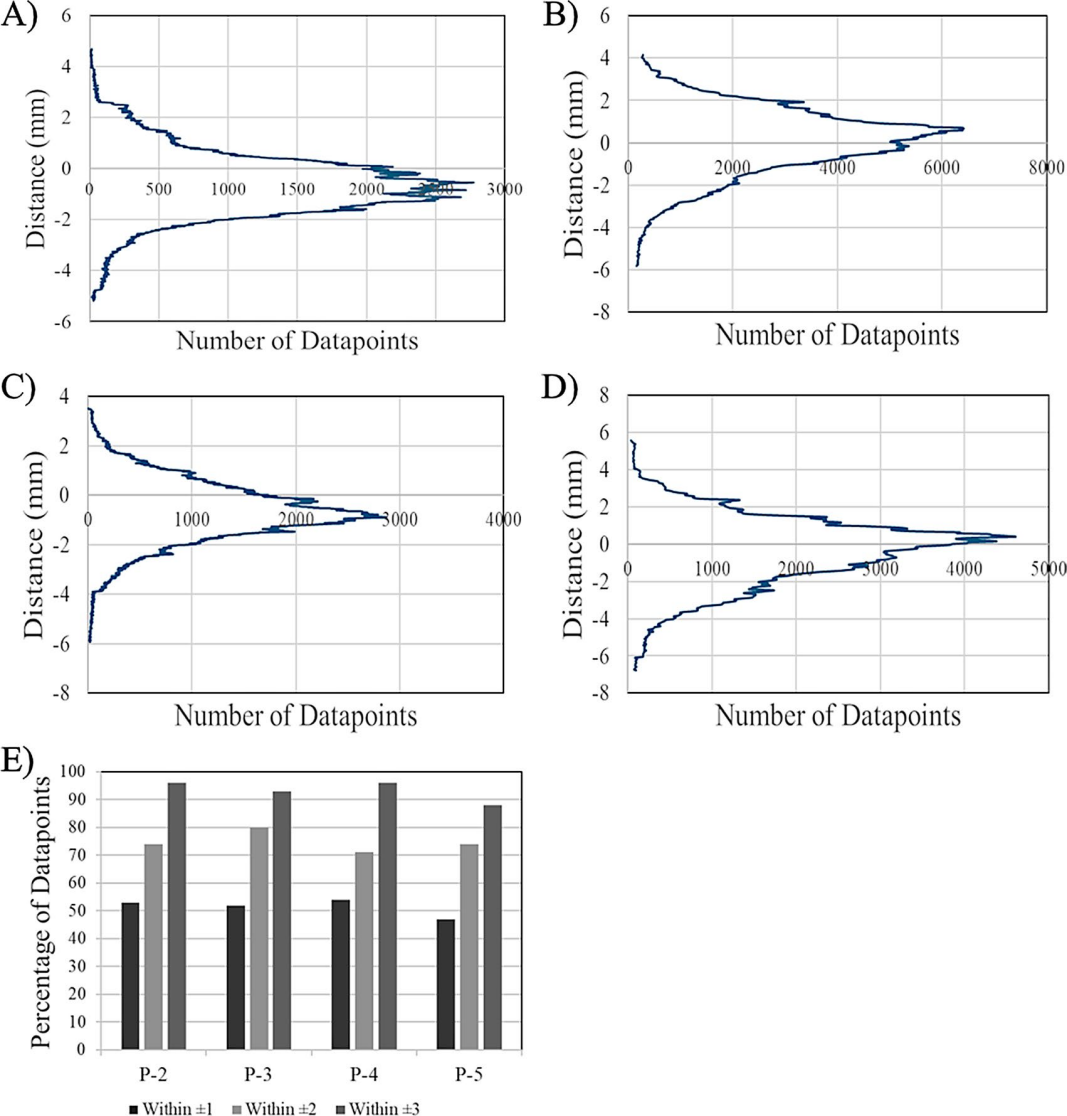
Nominal-actual comparison results for each ventricle model. Datapoint distribution of displacements from the 3D patient MRI model for (**A**) *P-2* ventricle model (**B**) *P-3* ventricle model (**C**) *P-4* ventricle model (**D**) *P-5* ventricle model. CAD comparison data was used to calculate percentages of data within specific tolerances. (**E**) Percentage of datapoints for each ventricle model that fell within ± 1-, 2-, and 3-mm from the original MRI model

**Fig. 13:**
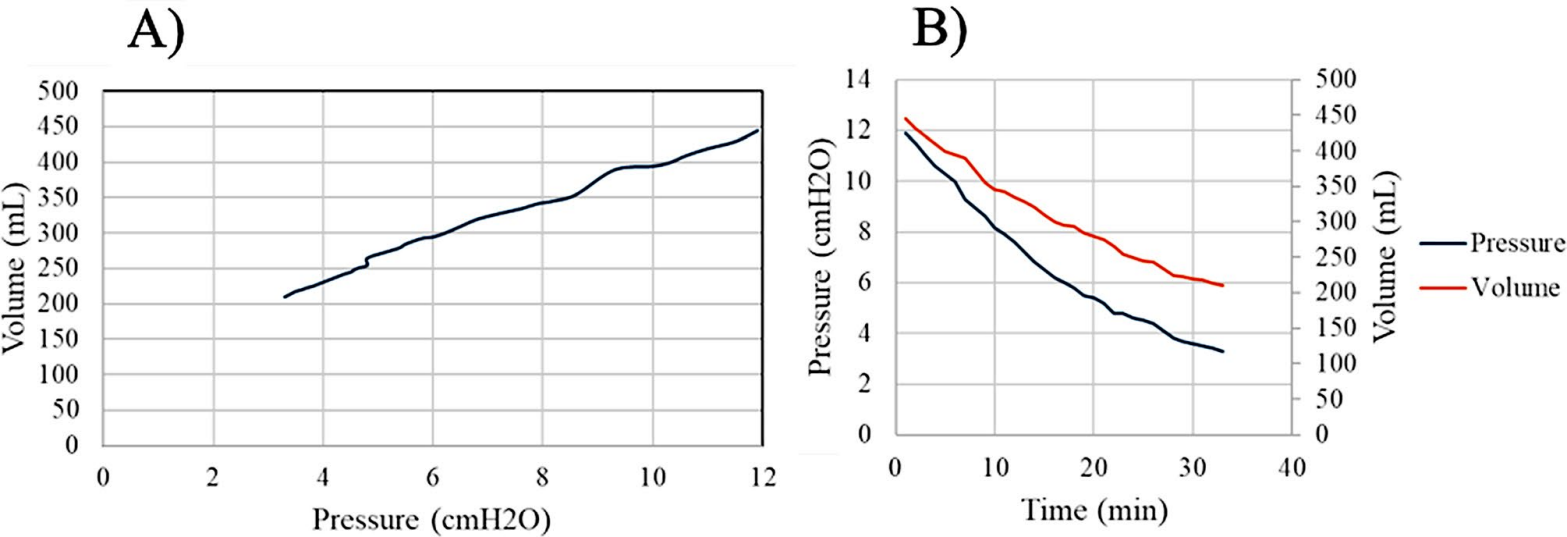
Pressure and volume changes over time and their relationship (**A**) Internal pressure and volume of the ventricle model for bulk compliance assessment. Linear regression analysis showed a strong positive correlation (*R*^*2*^ = 0.9932). (**B**) Change in internal ventricular pressure and volume during bulk compliance testing when the fluid was allowed to drain from the ventricle models

**Fig. 14:**
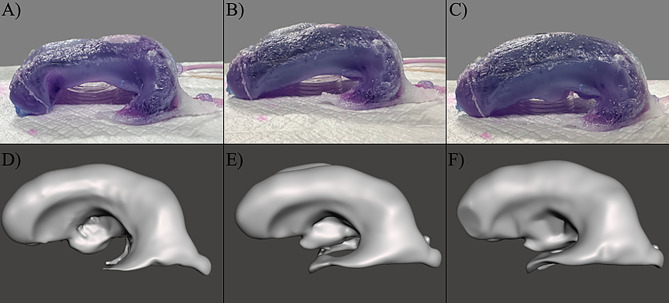
Qualitative profile comparison of the *P-5* ventricle model to its clinical counterpart. (**A**) The *P-5 (II)* manufactured ventricle model filled with approximately 198 mL of fluid (**B**) The ventricle model filled with approximately 275 mL of fluid (**C**) The ventricle model filled with approximately 321 mL of fluid (**D**) The *P-5 (III)* 3D ventricle model derived from a third consecutive MR image with a calculated internal volume of 198 mL (**E**) The *P-5 (II)* 3D ventricle model derived from a second consecutive MR image with a calculated internal volume of 275 mL (**F**) The *P-5 (I)* 3D ventricle model derived from a first of three consecutive MR image with a calculated internal volume of 321 mL. The volumes of fluid in each ventricle model corresponded to the volumes of the patient’s lateral and third ventricles at the time of scanning, calculated from MR images

The largest differences from the MRI model were observed in the *P-3* ventricle model resulting from differences in diameter measurements (Figs. 9B, 10B and 11). For both frontal horn diameter and Evan’s Index measurements, the manufactured ventricle model was greater than the clinical value by 19% due to an 8 mm difference between measured and clinical values. While the occipital horn diameter and FOHR measurements fell within ± 5% of clinical values, it exhibited the greatest percent difference compared to other ventricle models (OHD: -5% from clinical, FOHR: +3% from clinical). Between all ventricle models and consistent with *P-0* and *P-1* ventricle models, frontal horn diameter measurements were consistently greater than clinical and occipital horn diameter measurements were consistently less than clinical values. Nominal-actual comparison data of *P-2*,* P-3*,* P-4* and *P-5* ventricle models compared to their respective MRI models is displayed in Fig. 12 (S. Figures 11–14). A gaussian distribution was observed for all ventricle model displacements. Between all models, mean datapoint displacements all fell within ± 0.70 mm from their 3D MRI models. Similar to the *P-0* and *P-1* model replicates discussed in the verification process, ventricle models tended to skew smaller than the 3D MRI model. The smallest mean displacement was observed in the *P-3* ventricle model being 0.03 ± 1.65 mm (Fig. 12B, S. Figure 12). The largest mean displacement observed was in the *P-4* ventricle model being − 0.69 ± 1.27 mm (Fig. 12C, S. Figure 13). This relatively larger displacement with a heavy negative skew could be attributed to portions of the negative ventricle model used in 3D scanning not being fully developed (Fig. 8C). Percent datapoint displacements indicated how closely each scanned ventricle model geometry compared to their respective MRI model (Fig. 12E). The *P-2*, *P-3*, and *P-4* models showed that 52–54% of datapoints were within ± 1 mm of their MRI, while the *P-8* model only had 47% of all datapoints within the same displacement. 70–80% of datapoints fell within ± 2 mm displacement from their respective MRI model. Lastly, the *P-2*, *P-3*, and *P-4* showed 93–96% of all datapoints falling within ± 3 mm of their respective MRI. Again, *P-5* was a low-end outlier with just 88% of datapoints being in the same displacement.

### Anatomically accurate shifts in volume – an indirect test for bulk compliance capacity

A fully developed, sealed, and hollow ventricle model was successfully created with silicone rubber within a HIPS mold of the *P-5 (m)* MRI model. A sealed inlet and outlet were successfully created and no leakage was observed throughout the entire compliance testing process. Figure 11 displays the results from the bulk volume-pressure testing process. Linear regression analysis indicated that the coefficient of determination (*R*^*2*^) between for pressure and volume compared to time was 0.9788 and 0.9593, respectively (Fig. 13A). Further, the *R*^*2*^ between pressure and volume was 0.9932, indicating a positive linear correlation (Fig. 13B). Using the definition of compliance, a compliance of $\:27.33\:\frac{mL}{cm{H}_{2}O}$ was calculated for the silicone rubber ventricle model. Failure testing revealed that the silicone rubber walls ruptured at an internal volume of approximately 500 mL, with a maximum internal pressure of $\:13.8\:cm{H}_{2}O$ and failure being observed in the right frontal horn. Caliper measurements used to determine frontal horn and occipital horn diameters at internal volumes of 198 mL, 275 mL, and 321 mL showed that all diameter measurements were within ± 10% of the clinical value of the respective *P-5* model at that volume. All FOHR measurements fell within ± 5% of the clinical value. Evan’s Index ratios for the ventricle model when filled to 321 mL and 275 mL differed from the clinical MRI values by -3% and + 3%, respectively. However, when filled to 198 mL this difference increased to + 9% (Table 5). Qualitative comparisons were made in Fig. 14 by displaying images of the ventricle model when filled to each clinical volume (Fig. A, Fig. B, Fig. C) and pairing them side-by-side with images of *P-5* (b), *P-5* (m), and *P-5* (a) ventricle models filled to the same volumes. Morphological changes were seen in tabletop ventricle model that are comparable to the ones seen in the 3D models of the *P-5* MR images.

**Table 5:** Experimental measurements of the *P-5* manufactured ventricle model at increasing volumes. Percent differences indicate the amount that experimental measurements differed from the patient’s clinical FHD, OHD, evan’s Index, and FOHR for each MRI

CSF Volume (mL)	Frontal Horn Diameter (mm)	Occipital Horn Diameter (mm)	Evan’s Index	FOHR
198	60 (+ 9%)	81 (-4%)	0.43 (+ 9%)	0.51 (+ 1%)
275	67 (+ 3%)	85 (-9%)	0.45 (+ 3%)	0.51 (-4%)
321	72 (-3%)	93 (-7%)	0.48 (-3%)	0.55 (-5%)

## Discussion

This study introduced the novel application of a rotational molding-based process to manufacture phantoms of the lateral and third ventricles using MRI data from hydrocephalic patients. Verification and validation activities utilized injecting polyurethane foam into the ventricular molds to create a representative of the internal space that was 3D scanned and quantified in capable software. Compliance testing was done to understand how the 3D structure of a ventricle model changed with volume in relation to the material. Verification and validation results demonstrated the capability to manufacture ventricle models that clinically and anatomically represent the original patient MRI they were derived from. Pressure-volume assessment results (an indirect measure of bulk compliance without viscoelastic compliance) demonstrated the compatibility of the ventricle models with low-throughput fluid systems and ability to dynamically change, congruent with clinical data. It may be possible following future refinement to use these ventricle models to study relationships between ventricular catheter placement and wall contact, further characterization of shunt failure, and the real-time in vitro assessment of biological interactions within this ventricular volume range. Further, a dynamic, anatomically accurate model of the ventricular system has the potential to assist clinicians in managing hydrocephalus, such as by guiding surgeons on the placement of ventricular catheters in challenging ventricular structures or acting as a prognostic tool to understand how a specific patient’s ventricles may change over time. Altogether, the methods explored here lay the groundwork for developing a capable and clinically accurate model of the human ventricular system that is useful for hydrocephalus research.

### Clinical and 3D scanning valuation of ventricular size suggest similarity to MRI

Clinical comparisons of the manufactured ventricle models to the original patient MRI pointed to the models being overall accurate representations. In most cases, the frontal and occipital horn diameters of the different ventricle models did not vary from the actual clinical measurements by more than ± 5%, resultant of only a few millimeter differences in measurements. The result of this was the Evan’s Index and FOHR values for the ventricle models matching closely with respective MRI values as well (Figs. 9 and 10). The only significant departures from clinical values were observed in the *P-0-3* (S. Figure 10) and *P-3* (S. Figure 12) models, which both showed greater than ± 10% difference from clinical frontal and occipital horn measurements, suggesting a need to confirm relative variance prior to testing of additional patient-specific models. The clinical valuation for all models indicated that the diagnostic criterion for hydrocephalus is met [22, 23]. Using this metric to create a clinically accurate benchtop model of the ventricles differs from previous studies. Other models of the ventricle system, either benchtop or computational, have typically been compared to other clinical parameters such as CSF flow patterns or other non-clinical criteria [14, 16, 17, 24]. Further, they seldom incorporate consideration for 3D structure and anatomy into the model. Accurate recapitulation of horn-diameters is principle to constructing an accurate benchtop model for ventricle morphology characteristics, especially when considering the relevance that horn dilation has in evaluating hydrocephalus and its relation to wall expansion. Incorporation of CSF flow patterns in our model warrants future investigation.

Many previous studies that explore ventricular morphology have been largely limited to computational methods [25–27]. Our use of nominal-actual comparison tools was necessary to accurately compare the ventricle models to their respective MRI that enhances our understanding of anatomical similarity past FHD and OHD. The mean displacement of all models from their respective MRI-derived 3D models falling within ± 1 mm strongly shows that the 3D structures of the ventricle models produced through our process closely resemble the actual MRI (Figs. 3, 4 and 12). Being within this range suggests that the overall shape and size of the ventricle models is similar to the MRI and regions that are displaced a significant amount are isolated structures.

Two prominent dissimilarities observed in all ventricle models compared to MRI are a tendency for the frontal horns to ‘flare’ and the caudal portions of the lateral ventricles to be underdeveloped (S. Figures 10–14). This frontal horn flaring would explain the overall trend of experimental frontal horn measurements for the manufactured ventricle models to consistently be greater than or equal to clinical measurements. Underdevelopment of caudal portions is a potential explanation for the nominal-actual comparison results indicating that many of the ventricle models are on average smaller than the MRI model, even though they are volumetrically similar. It is possible that these patterns arise from a potential limitation of the polyurethane expanding foam, or slight inconsistencies in how the silicone rubber coats the inner walls of the mold during rotational molding, and not apparent defects of the mold itself. Regardless, the mitigation of this pattern between ventricle models should be addressed in further refinements of this model.

### Heterogeneous structure recapitulation

**Fig. 15:**
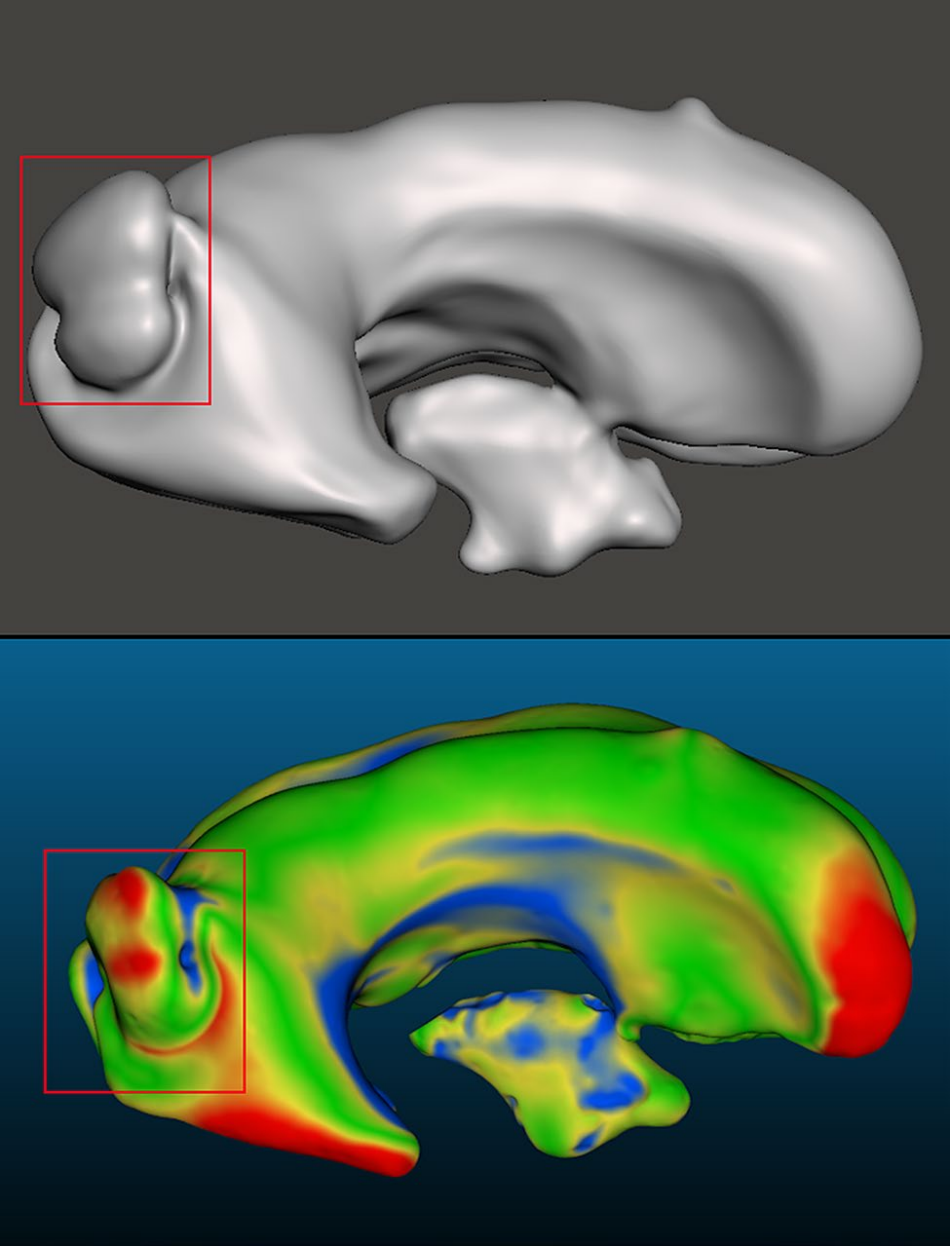
Right lateral ventricle images of the (top) *P-2* MRI derived model and the (bottom) 3D scan of the *P-2* ventricle model. Outlined with the red box is the same unique bulging structure present in the patient’s original MRI scan and the ability of our manufacturing process to recreate this same feature in the ventricle phantom. Other notable features visible include the notch on the cranial wall of the right lateral ventricle and a near-fully developed third ventricle

A notable capability of the manufacturing process observed was the ability to accurately recreate unique structures. This is well exemplified by the unique bulge present in the right occipital horn wall of the *P-2* MRI model being clearly reproduced in the manufactured ventricle model (Fig. 15). Other examples of this were observed in the *P-4* ventricle model, where large disproportionate sizes can be seen between frontal and occipital horns as well as an indenting notch reproduced from the patient MRI model. Previous studies have explored the relationship between ventricle structure and ventricular shunt failure related to over-drainage [9, 28]. We hypothesize that these or other unique structures that contribute to ventricular heterogeneity may influence the specific dynamics of the ventricle walls or the likelihood of shunt contact during expansion and contraction. This is a topic of future investigations.

### Verification of scalability and reproducibility

While a validation study alone could show that our methods accurately create a ventricle model from MRI data, verification was necessary to understand the reliability of the manufacturing methods. The low overall variances seen in the *P-0* model replicates were a strong indicator of the ventricle models being similar to each other and reproducible. This indication was even more prevalent when examining the clinical values of the *P-1* model replicates, where variances of σ^2^ < 0.333 were seen across all clinical categories (FHD, OHD, EI, FOHR). The larger but still relatively small variance seen in volumes for *P-1* model replicates (54.81–61.18 mL) warrants further investigation into the cause of within group differences. An even greater indication of reproducibility was seen in the nominal-actual comparison results for the *P-0* and *P-1* model replicates (Figs. 3, 4, 5, 6 and 7), which demonstrate all models to have similar percentage of datapoints between ± 1 mm, ± 2 mm, ± 1σ, and ± 2σ. Previous studies involving the use of ventricle models only employed purpose-built, single use setups which would likely be difficult to replicate on a meaningful scale for multiple different testing parameters [14, 15, 17, 29]. These data suggest that our methodology can reproduce multiple, different ventricle models with some heterogeneity and will allow us to explore hydrocephalus conditions in many different types of ventricular environments and a range of different dynamics. Continued improvement of the manufacturing process will allow for manufacturing of more complicated ventricle morphologies seen in patients with hydrocephalus that push the limits of what is achievable with the current setup. An important consideration in further development and manufacturing of these models is the propagation of uncertainty associated with any manufacturing process that can ultimately produce greater variability between model replicates. Regarding model replication with the current setup, limited adjustable variables are at play that introduce variation, those being the volume of silicone rubber injected and the speed of rotational molding. Both variables have a specific uncertainty/variance associated with them, whereas variables like the surface area of the mold, temperature, ambient pressure, material viscosity, and time under rotation are consistent and have negligible variance in the methodology presented here. However, for future iterations that may explore increasingly complex ventricle model molds or different materials, there is room for greater variation associated with each input variable that must be mitigated.

### Bulk compliance capacity assessment

The results of the compliance investigation produced intriguing results that suggest our ventricle models can replicate clinical expansion and contraction. Specifically, these data indicate how the ventricle model material itself contributes to compliance; the incorporation of a viscoelastic gel or a water surround as a brain substitute would also influence these responses. Evan’s Index and FOHR measurements for our ventricle model at each of the three volumes observed in the one patient over repetitive MRI closely match. The largest deviation seen in the Evan’s Index measurement when filled to 198 mL was still only a difference of 0.03 mm/mm, and all values fell within the diagnostic criteria for hydrocephalus. A limitation of the 3D scanning technique employed is the difficulty of producing accurate scans of soft and deformable bodies like our ventricle models. Potential for error is introduced when relying on caliper measurements to determine frontal horn and occipital horn diameters. This should be explored to better assess their accuracy against clinical measurements. Visually, the greatest expansion was seen in the frontal and occipital horns while the least expansion was seen near the temporal horns. which is consistent with the MRI results from the *P-5* patient tested (Fig. 14). Additionally, prior work has explored computational methods to map ventricle expansion patterns from MRI datasets and the results from the compliance testing of the ventricle models appear to be aligned with results from this previous study [25].

While the results of our compliance testing and quantification of the fluid filled *P-5* ventricle model suggested that expansion and contraction of the model was similar to physiologic observations, it is still unclear whether the amount of expansion observed would be clinically accurate across all ventricle models. The overall expansion of the model with increased volume is related to the compliance of the silicone rubber. The measured material compliance of $\:27.33\:\frac{mL}{cm{H}_{2}O}$ is significantly higher than reported intracranial compliance values, while the failure pressure of our model is within low-end values for physiological ICP ranges (9.52–20.39 $\:\frac{mL}{cm{H}_{2}O}$), but far below hydrocephalic ICP values exceeding 27.00 $\:\frac{mL}{cm{H}_{2}O}$ [30]. The discrepancies seen between compliance and pressure measurements of our model and physiological values is a major limitation of the ventricle model in its current iteration that should be addressed. These measured differences are a clear result of the significant difference in material properties of silicone rubber versus brain tissue, with a difference of several orders of magnitude between them. Previous studies on human brain tissue samples post-mortem suggest an average elastic modulus of 1.895 ± 0.592 kPa for white matter, the primary constituent of brain tissue [31]. Compared to the material properties for the chosen silicone rubber in this study (Table 2), the elastic moduli differ by a minimum factor of 52. The differences in material properties are further complicated by observed differences between white matter and gray matter, changes to the shear and viscoelastic properties observed in hydrocephalic patients, and the differing viscoelastic properties of silicone rubber and brain tissue [31–34]. These significant differences exemplify the difficulty at creating anatomically and physiologically accurate in vitro models of such dynamic systems. Potential possibilities for future work with our ventricle models could explore different materials with properties approaching brain tissue. Continuing in that direction, special importance should be placed on ensuring the biocompatibility of that material, were it to be possible to introduce live cells, blood, or protein into the interior of the ventricle model to expand the capabilities of an in-vitro ventricle model system. This topic is especially important to consider in modeling the ventricular system as a research tool to understand the relationship between morphological characteristics and ventricular catheter failure.

## Conclusions

In this study, a novel manufacturing method showed the capability of producing anatomically accurate MRI-driven ventricle models from a dataset of different patients with hydrocephalus. In addition to their anatomical accuracy, the ventricle phantoms were shown to respond dynamically to changes in internal fluid volume and pressure similar to clinical observations. The creation of these models is foundational to the future development of a robust in-vitro model of the ventricular system that can be a research tool for hydrocephalus. Future work with this manufacturing process will be aimed at improving the current process to improve results compared to clinical data, and incorporation of additional components to construct an encompassing model of the ventricular system.

## Supplementary Information

Below is the link to the electronic supplementary material.

Supplementary Material 1

## Data Availability

Data is provided within the manuscript or supplementary information. Further details are available through the corresponding author.

## Abbreviations

CSF: Cerebrospinal Fluid
EI: Evan’s Index
FH: Frontal Horn(s)
FHD: Frontal Horn Diameter
FOHR: Frontal-Occipital Horn Ratio
HIPS: High-impact Polystyrene
IRB: Internal Review Board
ISD: Inner Skull Diameter
MRI: Magnetic Resonance Imaging
OH: Occipital Horn(s)
OHD: Occipital Horn Diameter
QOL: Quality of Life
ROI: Region of Interest
STL: Standard Tesselation Language
VP: Ventriculoperitoneal
