# Supplementary material for: A novel method of fabricating dynamic, heterogenous benchtop lateral and third ventricle phantoms from MRI of hydrocephalus patients: a verification and validation study
Source: Fluids Barriers CNS. 2026 Jan 30;23:20. doi: 10.1186/s12987-025-00742-w (PMC12860057; doi:10.1186/s12987-025-00742-w)
Supplement: Supplementary file 1 — Supplementary Material 1 [file 12987_2025_742_MOESM1_ESM.docx]

**
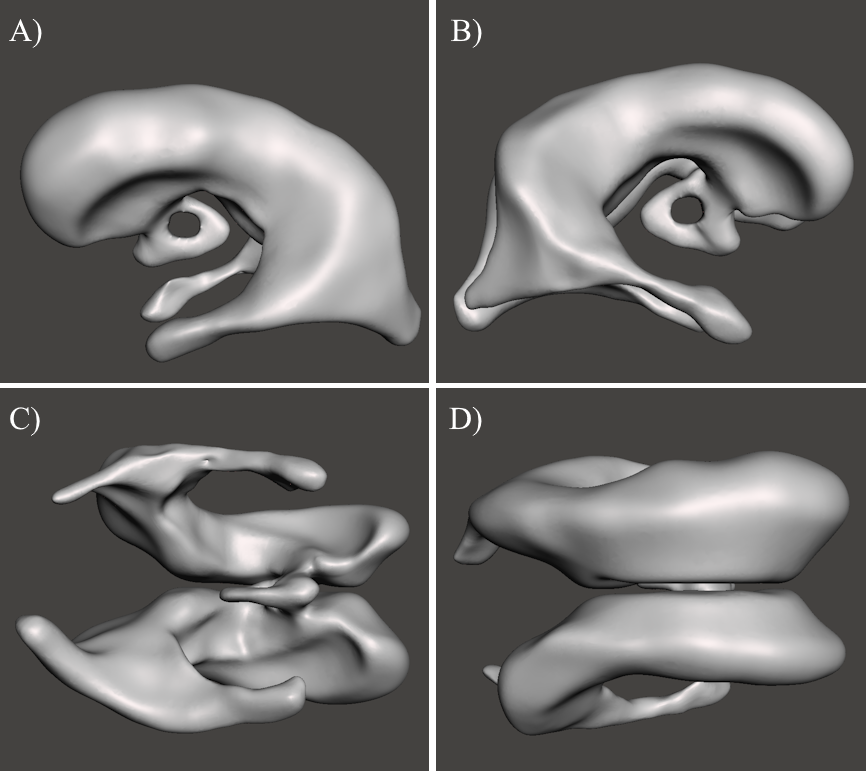
**

**S. Figure 1: Multi-angle views of the 3D *P-0* MRI model**. (A) Left lateral (B) Right lateral (C) Caudal (bottom-up) (D) Cranial (top-down) views of the MRI-derived 3D model from the *P-0* patient.

**
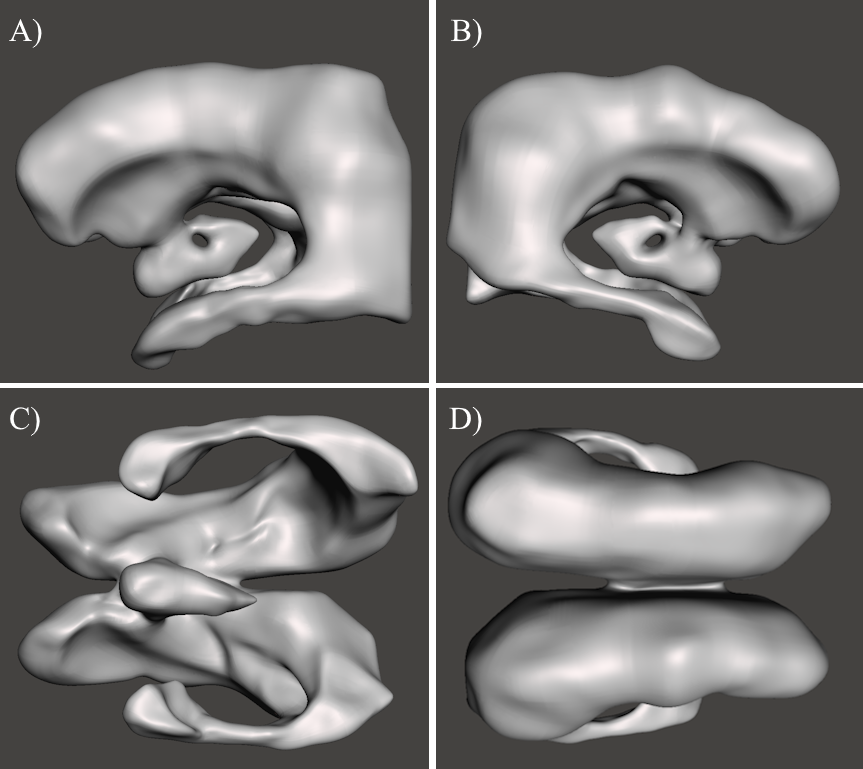
**

**S. Figure 2: Multi-angle views of the 3D *P-1* MRI model.** A) Left lateral (B) Right lateral (C) Caudal (bottom-up) (D) Cranial (top-down) views of the MRI-derived 3D model from the *P-1* patient.

**
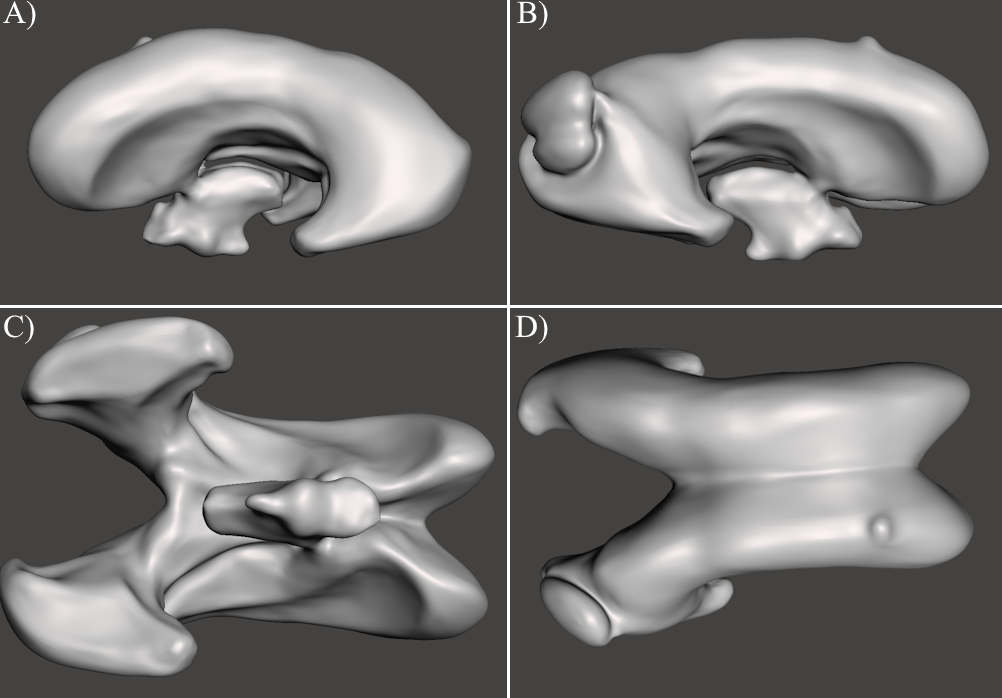
**

**S. Figure 3: Multi-angle views of the 3D *P-2* MRI model**. (A) Left lateral (B) Right lateral (C) Caudal (bottom-up) (D) Cranial (top-down) views of the MRI-derived 3D model from the *P-2* patient.

**
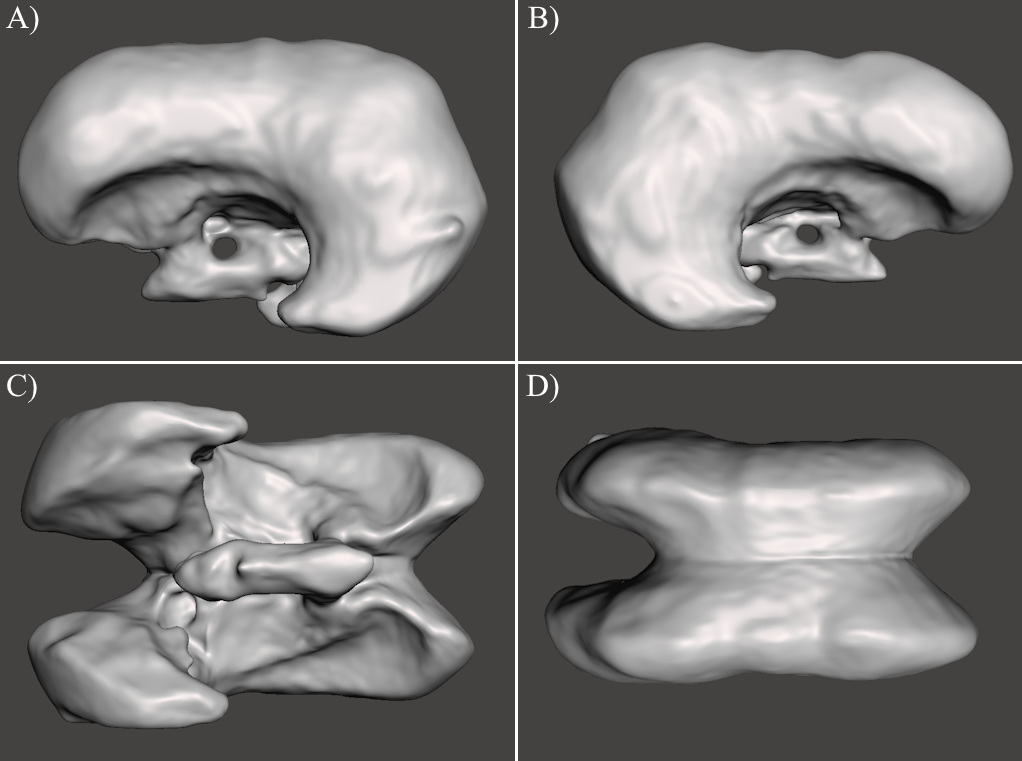
**

**S. Figure 4: Multi-angle views of the 3D *P-3* MRI model**. (A) Left lateral (B) Right lateral (C) Caudal (bottom-up) (D) Cranial (top-down) views of the MRI-derived 3D model from the *P-3* patient.

**
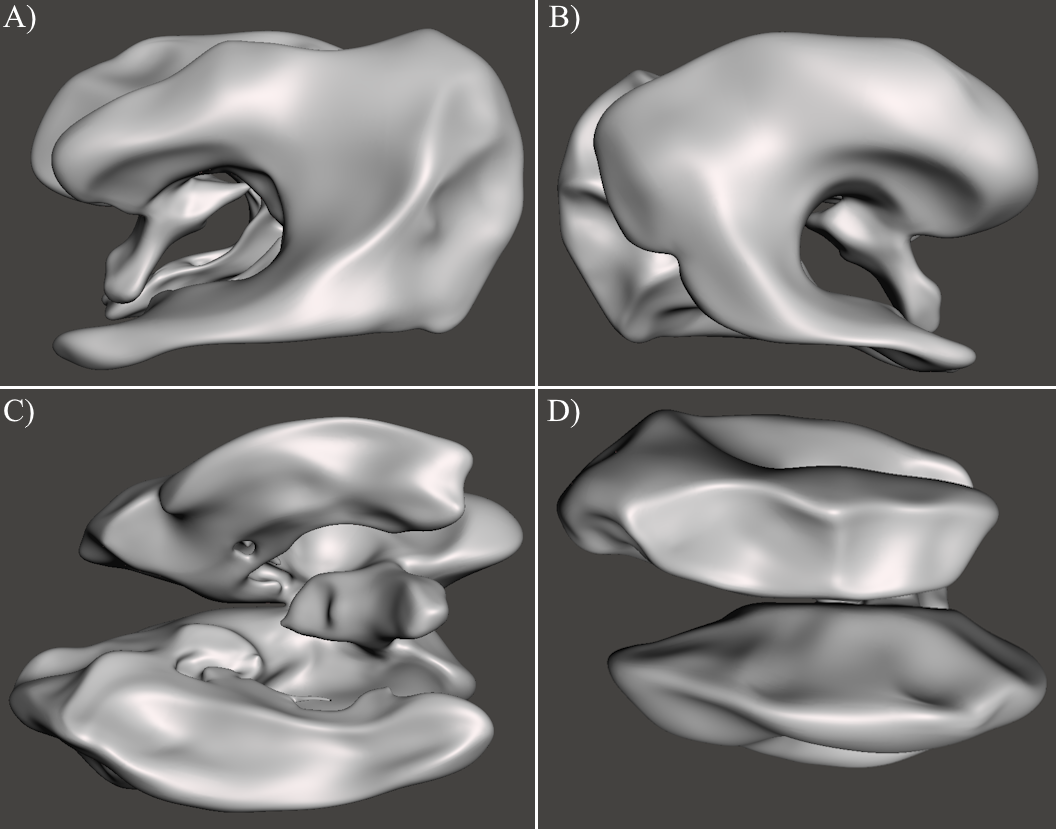
**

**S. Figure 5: Multi-angle views of the 3D *P-4* MRI model**. (A) Left lateral (B) Right lateral (C) Caudal (bottom-up) (D) Cranial (top-down) views of the MRI-derived 3D model from the *P-4* patient.

**
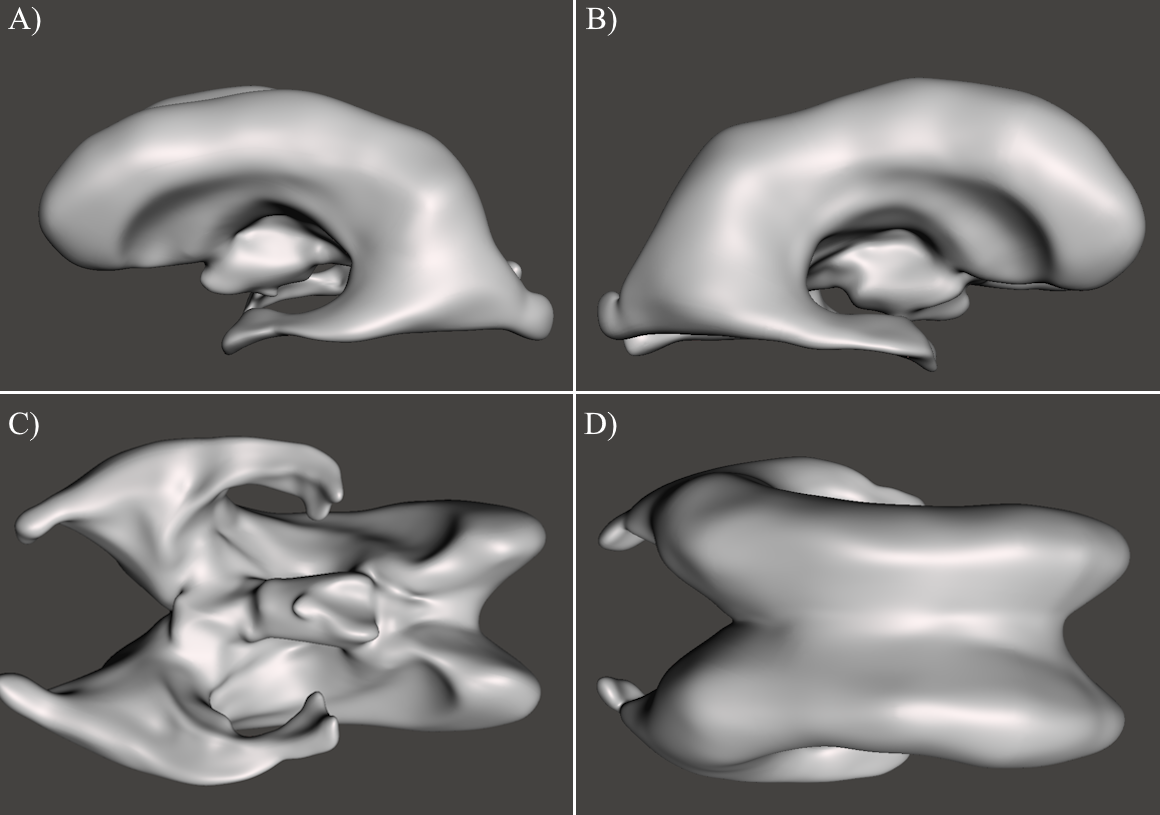
**

**S. Figure 6: Multi-angle views of the 3D *P-5 (II)* MRI model**. (A) Left lateral (B) Right lateral (C) Caudal (bottom-up) (D) Cranial (top-down) views of the MRI-derived 3D model from the *P-5* patient.

**
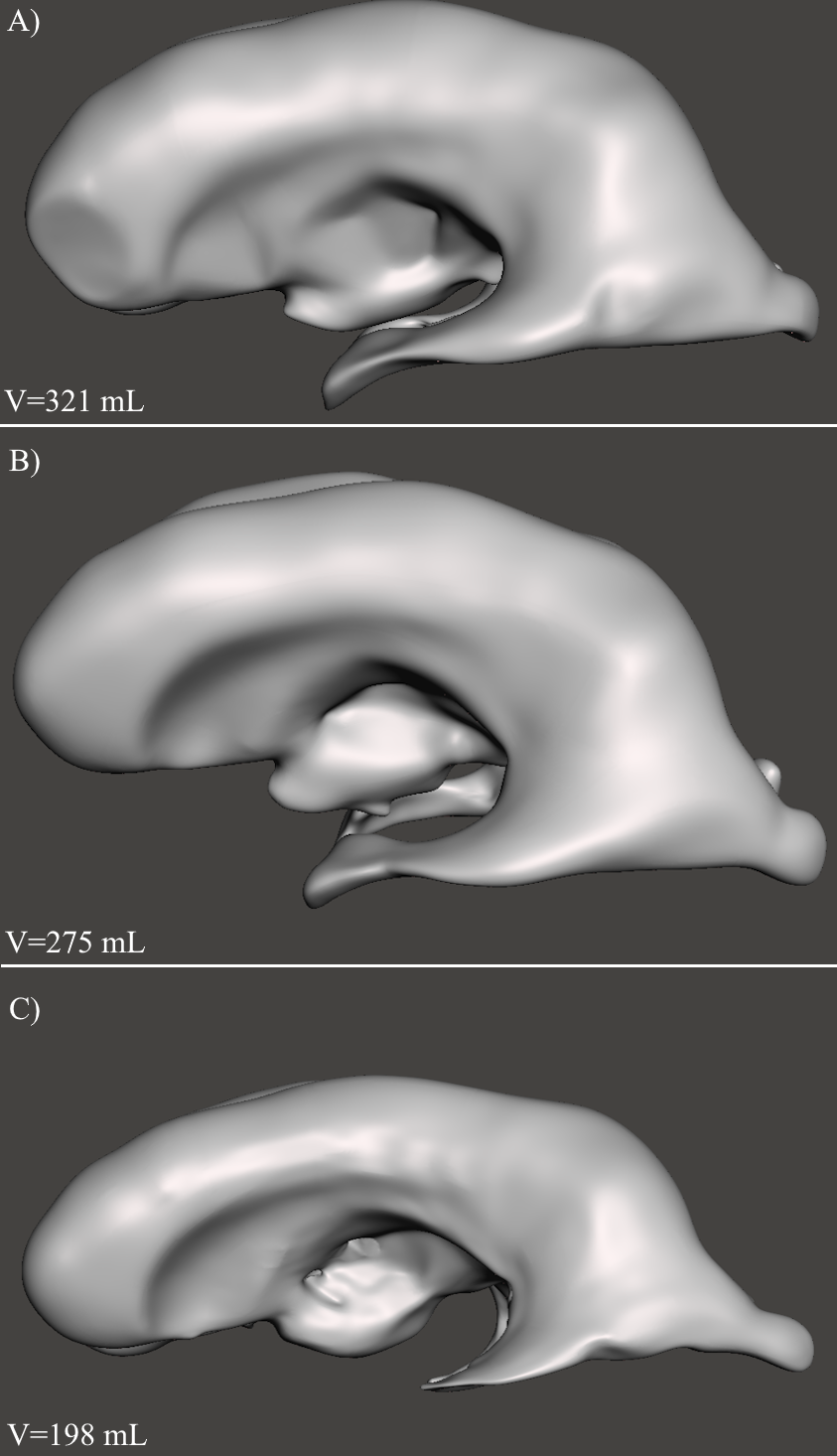
**

**S. Figure 7: MRI-derived 3D models of the *P-5 (I), (II),* and *(III)* scans.** Showcase of the 3D MRI model of the *P-5* patient displaying volumetric and morphological changes between the ventricles in repeat model scans. The internal CSF volume of the patient’s ventricles decreased with each consecutive scan I-III, leading to clinical CSF volumes of 321 mL, 275 mL, and 198 mL, respectively.

**
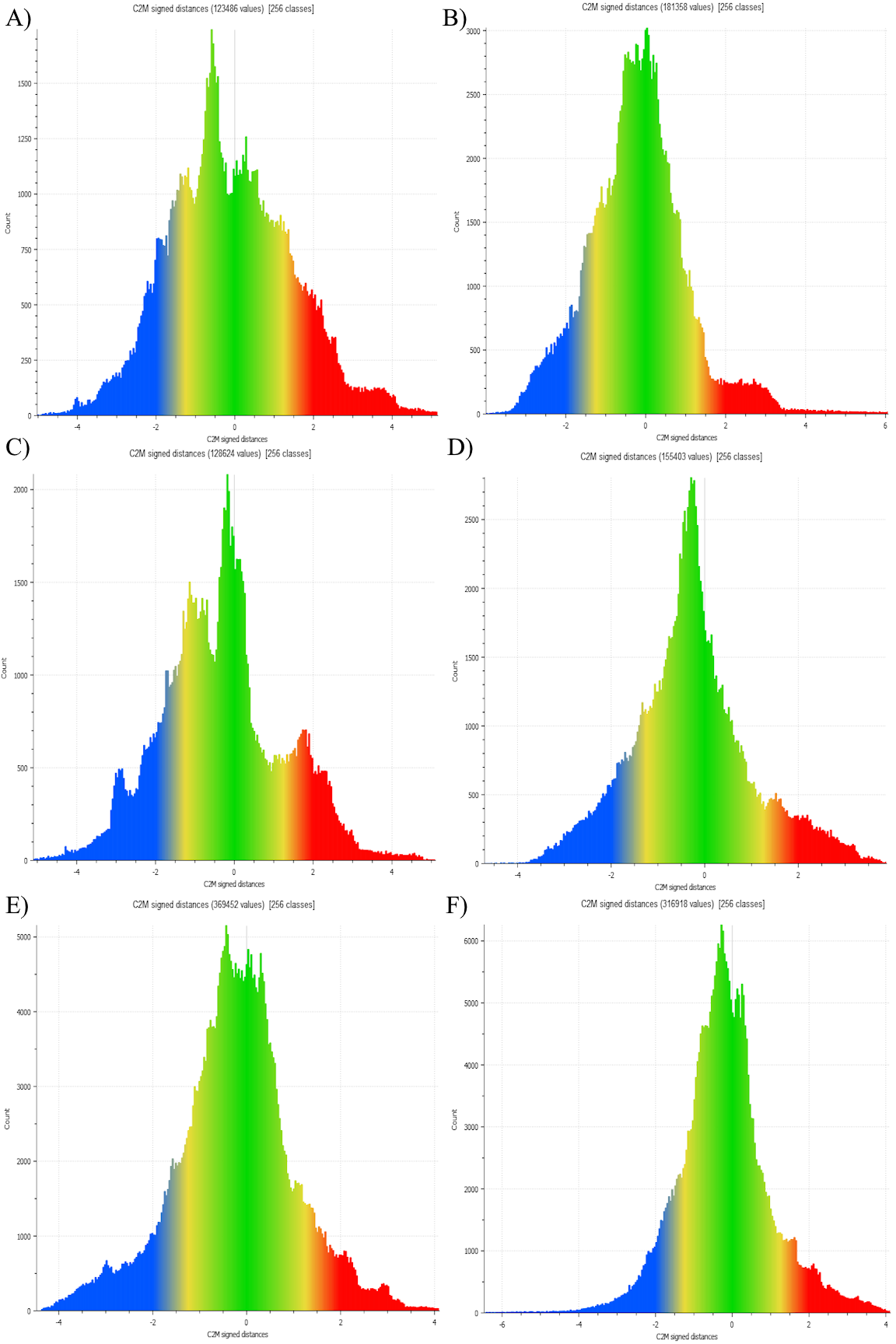
**

**S. Figure 8: Raw histogram data with nominal-actual comparison color scaling for *P-0* ventricle model replicates.** (A) *P-0-1* (B) *P-0-2* (C) *P-0-3* (D) *P-0-4* (E) *P-0-5* (F) *P-0-6*. ‘C2M Signed Distances’ is the displacement of the datapoint on the 3D scanned ventricle model from the equivalent datapoint on the patient MRI model, measured in millimeters.

**
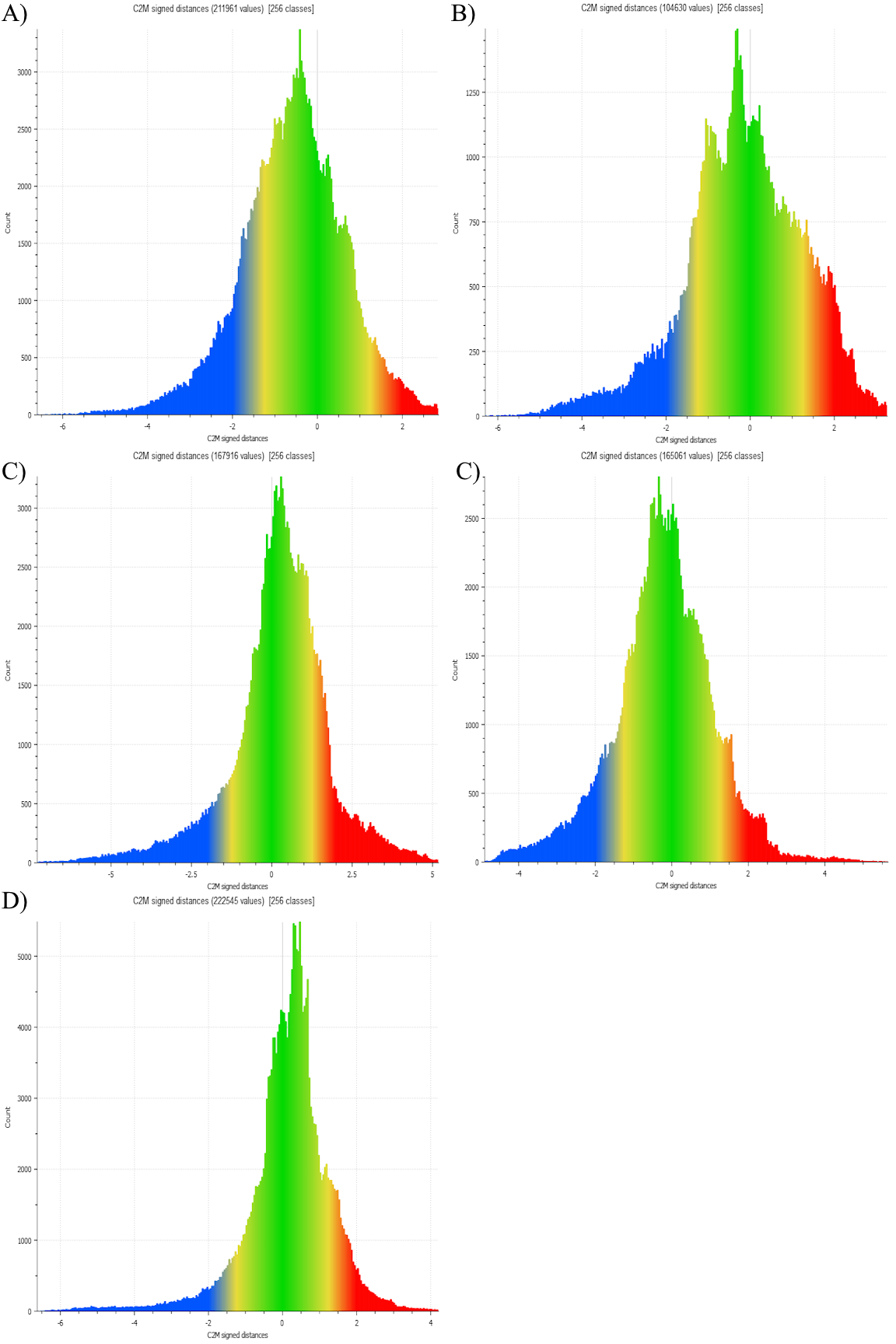
**

**S. Figure 9: Raw histogram data with nominal-actual comparison color scaling for *P-1* ventricle model replicates.** (A) *P-1-1* (B) *P-1-2* (C) *P-1-3* (D) *P-1-4* (E) *P-1-5*

**
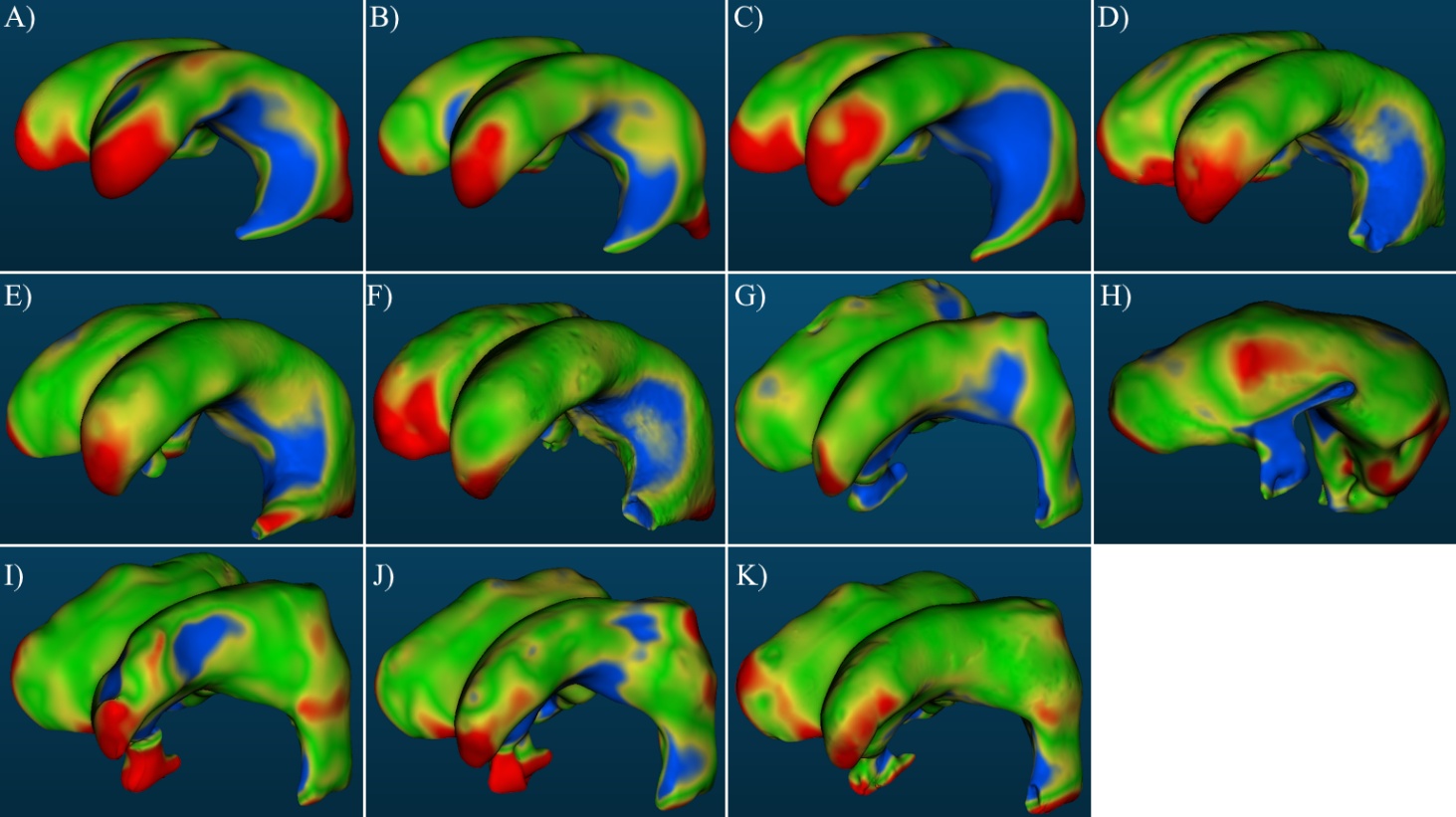
**

**S. Figure 10: Left frontal views of the *P-0* and *P-1* model replicates.** The applied colormap shows the results of the nominal-actual comparison and how well it compares to the patient MRI-derived 3D model. (A) *P-0-1* (B) *P-0-2* (C) *P-0-3* (D) *P-0-4* (E) *P-0-5* (F) *P-0-6* (G) *P-1-1* (H) *P-1-2* (I) *P-1-3* (J) *P-1-4* (K) *P-1-5*.

**
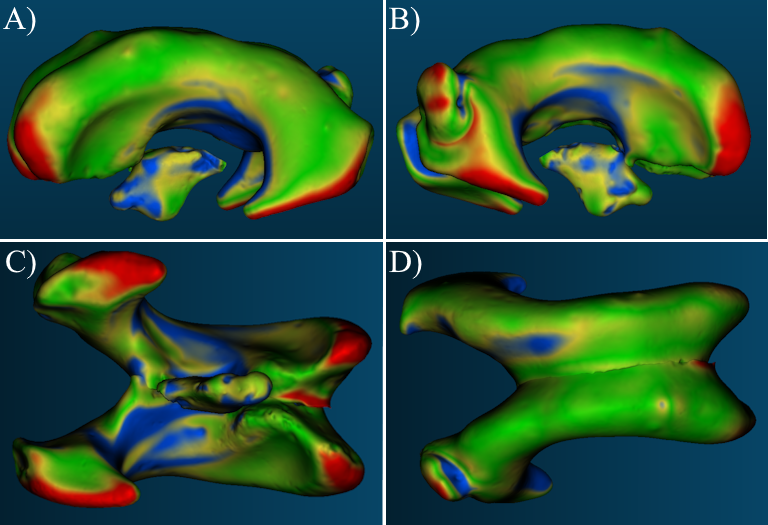
**

**S. Figure 11: Multi-angle views of the manufactured *P-2* ventricle model showing nominal-actual comparison results** (A) Left lateral (B) Right lateral (C) Caudal (D) Cranial

**
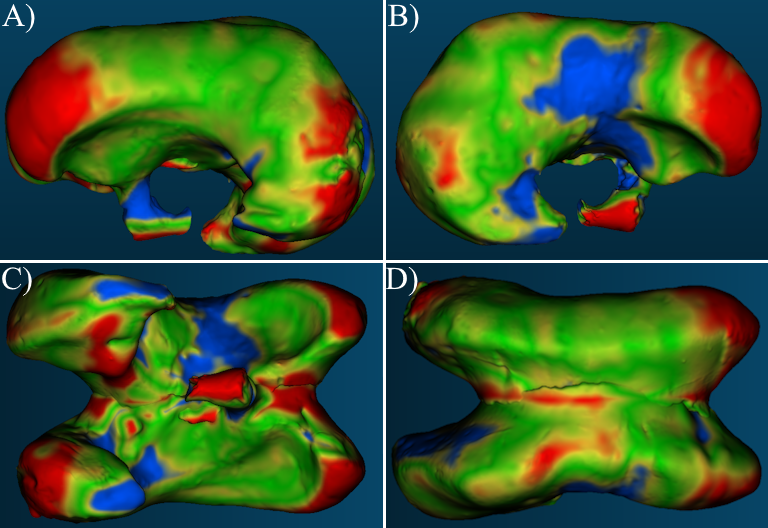
**

**S. Figure 12: Multi-angle views of the manufactured *P-3* ventricle model showing nominal-actual comparison results** (A) Left lateral (B) Right lateral (C) Caudal (D) Cranial

**
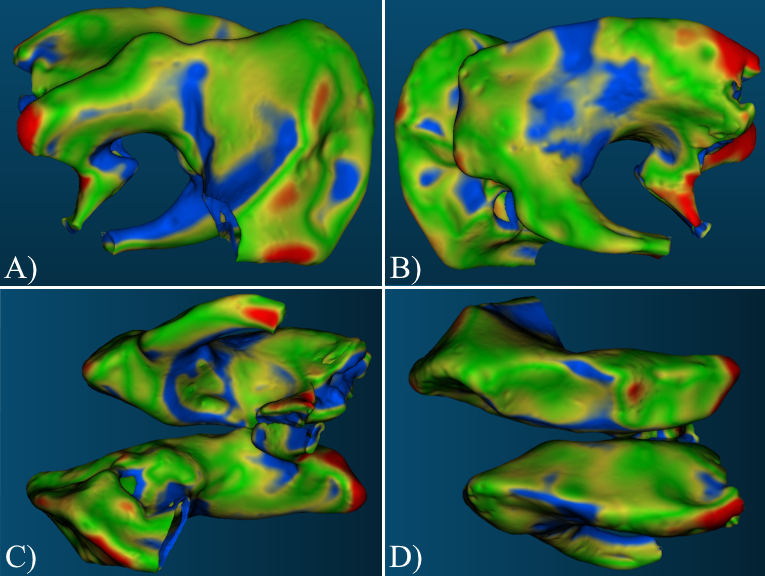
**

**S. Figure 13: Multi-angle views of the manufactured *P-4* ventricle model showing nominal-actual comparison results** (A) Left lateral (B) Right lateral (C) Caudal (D) Cranial

**
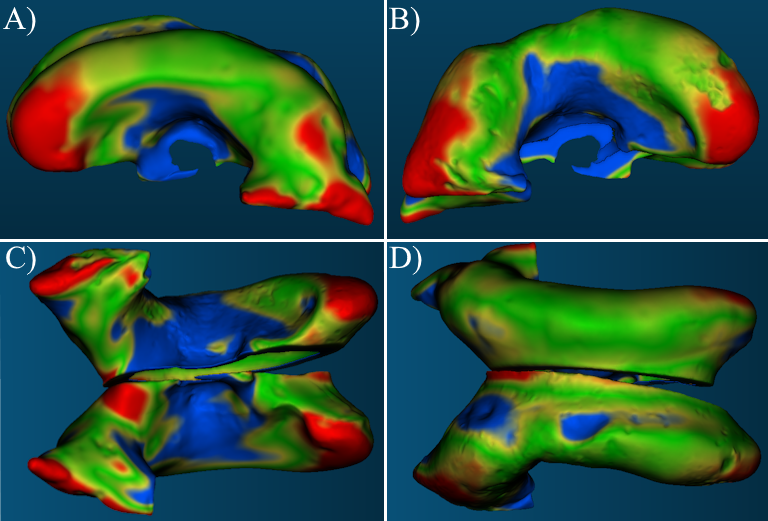
**

**S. Figure 14: Multi-angle views of the manufactured *P-5 (II)* ventricle model showing nominal-actual comparison results** (A) Left lateral (B) Right lateral (C) Caudal (D) Cranial
